# APC24-7, a covalent combination of boronic acid and chelator moieties, restores β-lactam efficiency against metallo-β-lactamase-producers

**DOI:** 10.1128/msphere.00418-25

**Published:** 2025-12-03

**Authors:** Rebekka Rolfsnes Hovd, Åsmund Kaupang, Pål Rongved, Geir Kildahl-Andersen, Knut Tormodssønn Hylland, Ragnar Hovland, Ole Andreas Løchen Økstad, Hanne Cecilie Winther-Larsen, Christopher Frøhlich

**Affiliations:** 1 Adjutec Pharma, Oslo, Norway; 2 Department of Pharmacy, University of Oslo 6305, Oslo, Norway; 3 Kappa Solutions, Oslo, Norway; 4 Department of Pharmacy, UiT The Arctic University of Norway 8016, Tromsø, Norway; University of Galway, Galway, Ireland

**Keywords:** antibiotic resistance, β-lactams, metallo-β-lactamases, serine β-lactamases, β-lactamase inhibitors, chelators, taniborbactam

## Abstract

β-Lactam/β-lactamase inhibitor combinations have significantly improved treatment outcomes for infections caused by serine β-lactamase (SBL)-producing pathogens. However, the continued emergence and spread of metallo-β-lactamases (MBLs), for which no clinically approved inhibitors currently exist, poses a serious threat to the long-term effectiveness of β-lactam-based therapies. To bridge this therapeutic gap, the boronic acid transition state analog, taniborbactam (Venatorx Pharmaceuticals), was developed, targeting SBLs and widespread MBLs such as NDM-1 and VIM-2. However, taniborbactam-escape variants have been detected among various MBL enzymes, including members of the NDM and IMP families. Here, we explored whether covalently combining two complementary inhibitor structures, a boronic acid transition state analog and a dipicolyl ethylenediamine-based metal chelator, can restore β-lactam susceptibility in MBL-producing bacterial strains, including taniborbactam-escape variants. APC24-7 successfully sensitized clinical isolates of SBL- and MBL-producing *Klebsiella pneumoniae* and *Escherichia coli* to meropenem. While APC24-7 demonstrated similarities in resensitization behavior to taniborbactam against a wide range of isogenic *E. coli* expressing single SBLs, APC24-7 reversed NDM-9- or IMP-26-mediated meropenem resistance more efficiently. To investigate the potential role of the chelator motif in the MBL inhibition of APC24-7, susceptibility tests were conducted with an excess of exogenous Zn²^+^. APC24-7-mediated resensitization remained unaffected in the presence of Zn²^+^ for strains producing NDM-1 and VIM-2. However, its ability to reverse NDM-9- and IMP-26-mediated meropenem resistance was attenuated upon Zn²^+^ supplementation. These findings demonstrate that combining functionally complementary chemical structures, such as chelators and boronic acids, can aid in expanding the resensitization ability of existing β-lactamase inhibitors.

**IMPORTANCE:**

The ability of bacteria such as *Klebsiella pneumoniae* and *Escherichia coli* to circumvent antimicrobial chemotherapy has become a global public health crisis. The high prevalence of β-lactamase enzymes capable of rendering our most prescribed antibiotics, the β-lactams (BLs) inactive, has left us with few available treatment options against infections caused by these bacteria. The use of small molecules that inhibit especially serine β-lactamases has substantially prolonged the lifetime of BL antibiotics. Yet, most clinically available inhibitors either do not possess or have limited ability to reverse resistance conferred by metallo-β-lactamase (MBL) enzymes. Combining chelator and transition state analog technology, our hybrid compound restores the effectiveness of BL antibiotics in cases of resistance conferred by both serine β-lactamases (SBLs) and MBLs. Our approach of covalently combining a chelator with an existing SBL inhibitor scaffold offers a promising solution for managing life-threatening infections and prolonging the use of clinically available BLs.

## INTRODUCTION

Antimicrobial resistance (AMR) has emerged as a global health crisis of immense proportions and is estimated to be the direct cause of 1.14 million deaths in 2021, and approximately 4.71 million deaths associated with the occurrence of AMR (1). One key driver of AMR is the production of β-lactamases: enzymes that catalyze the hydrolysis of the core β-lactam (BL) ring structure in BL antibiotics and even in the “last-resort” carbapenems (2, 3). These enzymes are classified based on their mechanisms of action, where serine-β-lactamases (SBLs) utilize a nucleophilic serine residue, while the function of metallo-β-lactamases (MBLs) relies on the presence of up to two Zn²^+^ ions within their active sites. Further classification is based on amino acid sequence identity, with Ambler classes A, C, and D representing SBLs, and Ambler class B encompassing the diverse and heterogeneous group of MBLs (4–7). The expression of MBLs is particularly concerning, as these enzymes hydrolyze virtually all BL antibiotics, except for monobactams (7, 8).

Combination therapy consisting of a BL and a β-lactamase inhibitor (BLI) has successfully extended the lifetime of many BLs by resensitizing β-lactamase-expressing clinical isolates to these antibiotics (3, 9–12). The use of combination therapy has been particularly effective against pathogens harboring SBLs, with significant treatment success following the clinical introduction of inhibitors such as the diazabicyclooctane avibactam (Zavicefta, ceftazidime/avibactam) and the boronic acid-based compound vaborbactam (Vaborem, meropenem/vaborbactam) (3, 13–16). However, a major limitation of these SBL-specific inhibitors is the frequent co-expression of both SBLs and MBLs in clinical isolates, limiting the effect of these inhibitors (17–20).

**Fig 1:**
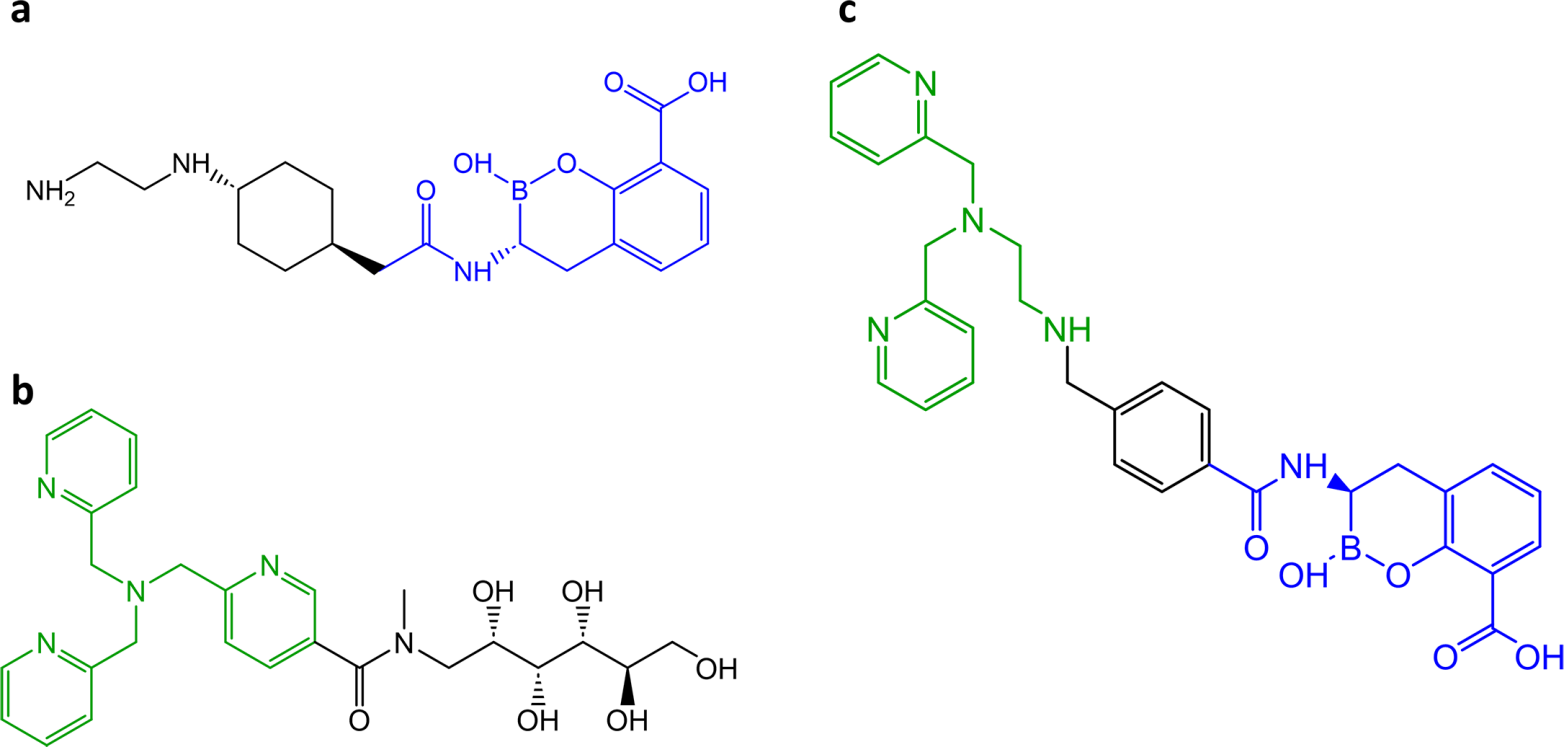
Chemical structures of BLIs*:* (**a**) taniborbactam, (**b**) APC148, and (**c**) APC24-7. APC24-7 derived from the boronic acid moiety (blue) of the dual MBL and SBL inhibitor taniborbactam and incorporates a dipicolyl ethylenediamine chelator inspired by the tris-(pyridylmethyl)amine chelator of APC148 (green).

While inhibitors that specifically target MBLs have been described in the literature, the structural diversity among enzymes has made it challenging to develop broad-spectrum inhibitors. While several metal-coordinating compounds have been reported, including carboxylate- and thiol-based structures, one of the more advanced candidates has been the bicyclic boronates, among which taniborbactam (Fig. 1a) has shown promising results (3). Taniborbactam is a boronic acid-based inhibitor acting as a transition state analog and has been developed to address the issue of co-expression of β-lactamases and shows promising inhibition of both SBLs and MBLs (21–23). The combination cefepime/taniborbactam has recently demonstrated inhibitory potential against clinically relevant enzyme families within SBLs (KPC and OXA-48-types), as well as within MBLs (VIM- and NDM-like) (22, 24–29).

Despite its general broad-spectrum inhibitory potential, published data reveal that taniborbactam exhibits limited activity against certain MBLs. This includes globally circulating IMP-type enzymes (e.g., IMP-1, IMP-4, and IMP-8) and specific NDM family variants (e.g., NDM-9 and NDM-30) in which a lower binding affinity (*K*_*i*_) and/or inhibitor activity (IC_50_) has been proposed to limit the effect of taniborbactam (21, 30–32). The emergence of taniborbactam-escape variants among clinical isolates may represent a potential obstacle to its broader clinical application (26, 32–35).

The lipophilic Zn^2+^ chelating structure tris-picolylamine (TPA) and its more hydrophilic variant dipicolyl ethylenediamine (DPED) have been shown to confer strong inhibitory effects toward a variety of MBLs (36–42). The previously described MBL inhibitor APC148 (formerly named ZN148, Fig. 1b) exhibits potent inhibitory activity against several MBL types, including NDMs, VIMs, and taniborbactam-escape variants of the IMP family (40). APC148 successfully restored meropenem susceptibility in over 98% of the 234 tested MBL-producing clinical Enterobacterales strains (IHMA collection) and showed no effect in strains expressing serine-type carbapenemases. APC148 apparently inhibits MBL activity in an irreversible manner, as it was shown that treatment and consequent removal of the inhibitor only led to a ~30% recovery of enzyme activity (40, 43). In addition, APC148 did not generate acute toxicity in mice at cumulative doses of up to 128 mg/kg and completed a clinical phase 1 single ascending dose (SAD) study (NCT06360640) (40).

We hypothesized that combining inhibitory moieties with complementary modes of action, such as a Zn^2+^-chelator moiety and a transition state analog structure, could yield a hybrid inhibitor with a broadened ability to resensitize MBL-mediated BL resistance. We developed and tested APC24-7, a compound that integrates the boronic acid structure of taniborbactam linked to a DPED-type chelator moiety through a benzoyl amide (Fig. 1c) (44). APC24-7, alongside taniborbactam, was evaluated against clinical and isogenic isolates expressing a diverse range of SBLs and MBLs, including variants inadequately inhibited by taniborbactam (NDM-9 and IMP-26). To evaluate the contributions of the DPED chelator and boronic acid structures, we examined the impact of exogenous Zn²^+^ on APC24-7’s ability to reverse MBL-mediated BL resistance. Our approach paves the way for the design of new and improved BLI scaffolds by harnessing chemical structures with complementary modes of action.

## RESULTS

### APC24-7 in combination with meropenem displays concentration-dependent sensitization of clinical bacterial isolates

APC24-7 represents a combination of the bicyclic boronic acid moiety used in taniborbactam linked with a Zn^2+^-chelator structure (DPED) and was synthesized through a multi-step process (Protocol S1). In brief, the chelator and boronic acid moieties in their protected forms were synthesized separately, yielding a chelator carrying a carboxylic acid group and a boronate ester with an α-amino group. These were coupled using a HATU-mediated amide coupling, yielding fully protected APC24-7. Deprotection using BCl_3_ and purification under alkaline conditions yielded the water-soluble disodium salt of APC24-7 (Fig. S1).

To evaluate the ability of APC24-7 to reverse β-lactamase-mediated BL resistance, 16 clinical isolates of *Escherichia coli* (*n* = 8) and *Klebsiella pneumoniae* (*n* = 8) were selected for analysis. These isolates were chosen based on the presence of β-lactamases acting on carbapenems and were categorized as SBL only (*n* = 4), MBL only (*n* = 9), and isolates co-expressing MBL and SBL enzymes (*n* = 3). The study aimed to include clinically relevant SBL carbapenemases such as KPC-2 (class A), OXA-48, and OXA-181 (class D), which are notorious for their widespread dissemination (45, 46). Similarly, strains harboring clinically significant MBLs, including VIM-, IMP-, and NDM-type enzymes, were included in the analysis (4, 7, 47). Given the high prevalence of SBLs in clinical isolates, 15 of the 16 tested strains also carried non-carbapenemases β-lactamase enzymes, such as TEM-, SHV-, CMY-, CTX-M-, OXA-types, and AmpC (Table S1).

To determine whether APC24-7 exhibits inherent antibacterial activity, the inhibitor was incubated with a selection of the clinical isolates at high concentrations (up to 906 µM/512 mg/L) (Table S2). The incubation with the inhibitor alone did not prevent bacterial growth up to the highest concentration tested, suggesting that APC24-7 does not possess antibacterial activity nor show any pleiotropic effects on the bacterial cells by itself below this concentration. Subsequently, we aimed to identify a potent β-lactam/APC24-7 combination capable of reversing β-lactamase-mediated BL resistance in our clinical isolates. All 16 isolates were exposed to increasing concentrations of meropenem (carbapenem), aztreonam (monobactam), ceftazidime and cefepime (cephalosporins), as well as amoxicillin (penicillin), both in the absence and presence of 57 µM (32 mg/L) of APC24-7. The effectiveness of these combinations was assessed based on their ability to resensitize the clinical isolates according to the clinical breakpoints (EUCAST clinical breakpoint table version 15.0, www.eucast.org).

APC24-7 successfully reduced the minimum inhibitory concentration (MIC) of meropenem in 12 out of 16 clinical isolates to the susceptible breakpoint category (MIC ≤2 mg/L) (Table 1). However, when combined with the other tested BLs, APC24-7 showed limited efficacy in reversing BL resistance, resensitizing only a small number of strains (aztreonam: 4; ceftazidime: 2; cefepime: 6; and amoxicillin: 0) (Table S3). Since meropenem/APC24-7 was the most potent BL/BLI combination, we further evaluated the efficacy of APC24-7 in combination with meropenem at lower inhibitor concentrations (28 and 14 µM, corresponding to 16 and 8 mg/L, respectively). Meropenem MICs recorded at these concentrations demonstrated reduced resensitization ability, with only 8 out of 16 isolates resensitized at 28 µM and 5 out of 16 at 14 µM (Table S4). These findings highlight that APC24-7’s ability to reverse meropenem resistance is highly concentration-dependent, with substantially diminishing effects at lower concentrations.

**TABLE 1:** The MICs of meropenem (MEM) alone, or in combination with APC24-7 or taniborbactam (TAN), against clinical isolates of *E. coli* and *K. pneumoniae.*

Bacteria	Isolate	Carbapenemase(s)	Other β-lactamases	MIC MEM (mg/L)		
				MEM only	APC24-7^*a*^	TAN^*a*^
Isolates producing SBL carbapenemases						
*E. coli*	KresCPE0097	KPC-2	NA^*b*^	1–2	<0.03	<0.03
*K. pneumoniae*	BAA1705	KPC-2	SHV-1, TEM, OXA-18	32	0.25	≤0.03
*E. coli*	KresCPE0348	OXA-48	CTX-M-24, TEM-1	>64	64	2
*K. pneumoniae*	KresCPE0385	OXA-48	CTX-M-15, SHV-1	16	16	0.5
Isolates producing MBL carbapenemases						
*E. coli*	BAA-2469	NDM-1	OXA-1, AmpC	32–64	≤0.03	0.06
*K. pneumoniae*	BAA-2146	NDM-1	SHV-1, CTX-M-1, TEM, OXA-1, AmpC	>64	0.12	2
*K. pneumoniae*	K66-45	NDM-1	SHV-11, CTX-M-15, OXA-1, OXA-9, TEM-1	32–64	0.06	0.25
*K. pneumoniae*	#29	NDM-1	SHV-11, CTX-M-15, OXA-1, CMY-6	64	0.5	0.25
*E. coli*	KresCPE0367	NDM-5	CTX-M-15, OXA-1	>64	2	0.25
*E. coli*	#36	NDM-7	CTX-M-15, OXA-1	>64	4	2
*K. pneumoniae*	K46-62	VIM-1	SHV-12, TEM-1	32–64	0.06	0.06
*E. coli*	#50	VIM-4	CTX-M-15, CMY-4, TEM-169	16–32	≤0.03	0.06
*E. coli*	#53	IMP-26	CTX-M-15, TEM-1	8–16	0.25	16
Isolates producing MBL and SBL carbapenemases						
*K. pneumoniae*	KresCPE0353	NDM-1, KPC-2	CTX-M-15, OXA-1, SHV-11	>64	0.25	0.25
*K. pneumoniae*	#22	NDM-1, OXA-181	SHV-11, CTX-M-15, OXA-1, TEM-1	>64	32	8
*E. coli*	KresCPE0372	NDM-5, OXA-181	CMY-42, TEM-1	>64	1	0.25

^*a*^ Inhibitor concentration was fixed at 57 μM.

^*b*^ NA, not applicable.

Next, we verified the molecular target of APC24-7 by subjecting a diverse set of purified MBLs (NDM-1, NDM-9, VIM-2, VIM-7, SHD-1, ECV-1, and MYO-1) (48), as well as SBL OXA-48, to enzyme inhibition kinetics (Table S5 and Fig. S2). Dose-response curves and calculated inhibitor potencies (IC_50_) revealed inhibition of both MBLs and SBLs with IC_50_ values ranging from 0.06 to 2.66 µM (pIC_50_ 7.3 to 5.6), validating these enzymes as molecular targets for APC24-7.

### APC24-7 and taniborbactam exhibit distinct inhibition patterns in clinical isolates

To assess whether the structural extension of the boronic acid moiety with the DPED chelator in APC24-7 affects the overall inhibitory spectrum compared to taniborbactam, meropenem MICs were recorded for each of the inhibitors at equimolar concentrations (57 µM) (Table 1). APC24-7 successfully reduced meropenem MICs below the clinical breakpoint (MIC ≤ 2 mg/L) for 12 out of 16 isolates. Exceptions included *E. coli* KresCPE0348 (OXA-48), *K. pneumoniae* KresCPE0385 (OXA-48), *E. coli #36* (NDM-7), and *K. pneumoniae* #22 (NDM-1 and OXA-181), where MIC values remained above the clinical breakpoint. In comparison, meropenem/taniborbactam sensitized 14 out of 16 isolates. As expected*, E. coli* #53 expressing a taniborbactam-escape MBL variant (IMP-26) did not respond to taniborbactam and hence meropenem resistance was not reversed. Similar to APC24-7, taniborbactam was unable to sensitize *K. pneumoniae* #22 (NDM-1 and OXA-181). However, unlike APC24-7, taniborbactam successfully sensitized *E. coli* KresCPE0348 (OXA-48), *K. pneumoniae* KresCPE0385 (OXA-48), and *E. coli #36* (NDM-7) to meropenem, highlighting a dissimilarity in the inhibition patterns of these two inhibitors.

Our findings from clinical isolates demonstrate that APC24-7 and taniborbactam exhibit distinct abilities to resensitize strains to meropenem. Nonetheless, the combination of the boronic acid with a pyridine-based chelator does not seem to substantially affect the resensitization effect conferred by this moiety. Since APC24-7 displayed an ability to reverse resistance to BLs other than meropenem, the discrepancy between APC24-7 and taniborbactam likely stems from mechanisms unrelated to β-lactamase production, for example, such as differences in uptake or efflux pathways (49).

### APC24-7 displays a broader spectrum of inhibition against MBL enzymes compared to taniborbactam

The clinical isolates used in this study likely express resistance mechanisms beyond β-lactamases. Therefore, we investigated the ability of APC24-7 and taniborbactam to restore BL activity in an isogenic system (Table S1). Single β-lactamase enzymes from Ambler class A (KPC-2 and CTX-M-15), class B (NDM-1, NDM-4, NDM-7, NDM-9, NDM-16, IMP-26, and VIM-2), and class D (OXA-48 and OXA-163) were cloned into a low-to-medium-copy-number vector (pA15 origin) (50, 51) and expressed in an isogenic *E. coli* strain (E. cloni 10G) (Table S1). MICs were recorded in the absence and presence of 9 µM of the inhibitors (corresponding to 4 mg/L of taniborbactam), which reflects the clinical screening concentration for taniborbactam resistance (22, 25). In addition to meropenem MICs, susceptibility to cefepime was also evaluated, given the completion of Phase III trials of the cefepime/taniborbactam combination (52, 53).

APC24-7 demonstrated a potent ability to restore meropenem susceptibility in isogenic strains producing MBLs (NDM-1, NDM-4, NDM-7, NDM-16, and VIM-2, with MIC reductions of 16- to 512-fold) and SBLs (KPC-2 with a 128-fold reduction and OXA-48 with a fourfold reduction) (Table 2). When combined with cefepime, APC24-7 reversed cephalosporin resistance by ≥16-fold in strains producing NDM-1, NDM-4, NDM-7, NDM-16, OXA-163, KPC-2, and CTX-M-15 (Table 2). The MICs for meropenem and cefepime in combination with APC24-7 were comparable to those with taniborbactam, with all differences being within a twofold range. However, at the tested inhibitor concentration (9 µM), neither APC24-7 nor taniborbactam exhibited any effect in combination with meropenem or cefepime against isogenic strains producing IMP-26 and NDM-9.

**TABLE 2:** The MICs of meropenem (MEM) and cefepime (FEP) alone, and in combination with APC24-7 and taniborbactam (TAN), respectively, in isogenic *E. coli* E. cloni 10G harboring β-lactamases from Ambler classes A, B, and D^*a*^

Strain^*b*^	Ambler class	MIC MEM (mg/L)			MIC FEP (mg/L)		
		MEM alone	APC24-7^*c*^	TAN^*c*^	FEP alone	APC24-7*^c^*	TAN^*c*^
MP 21-05 (*E. coli* E. cloni)	NA	0.03	0.03	0.03	0.008	0.008	0.008
MP 24-44 (KPC-2)	A	4	0.03	0.03	0.25	0.008	0.008
MP 24-80 (CTX-M-15)	A	NT	NT	NT	4	0.008	0.016
MP 30-63 (NDM-1)	B	32	0.06	0.12	8	0.03	0.06
MP 30-13 (NDM-4)	B	32	0.12	0.25	32	0.06	0.06
MP 30-20 (NDM-7)	B	32	0.12	0.06	16	0.06	0.06
MP 30-15 (NDM-9)	B	32	16	16	32	8	16
MP 30-22 (NDM-16)	B	16	0.06	0.12	4	0.03	0.03
MP 30-57 (VIM-2)	B	0.5	0.03	0.03	0.03	0.008	0.008
MP 30-58 (IMP-26)	B	4	4	4	0.25	0.12	0.25
MP 21-01 (OXA-48)	D	0.25	0.06	0.03	NT	NT	NT
MP 29-27 (OXA-163)	D	NT	NT	NT	0.25	0.016	0.016

^*a*^ NT: not tested since β-lactamase is not expected to decrease the susceptibility toward the BL (54–56).

^*b*^ Species of all constructs is *E. coli*.

^*c*^ Inhibitor concentrations were fixed at 9 μM.

**Fig 2:**
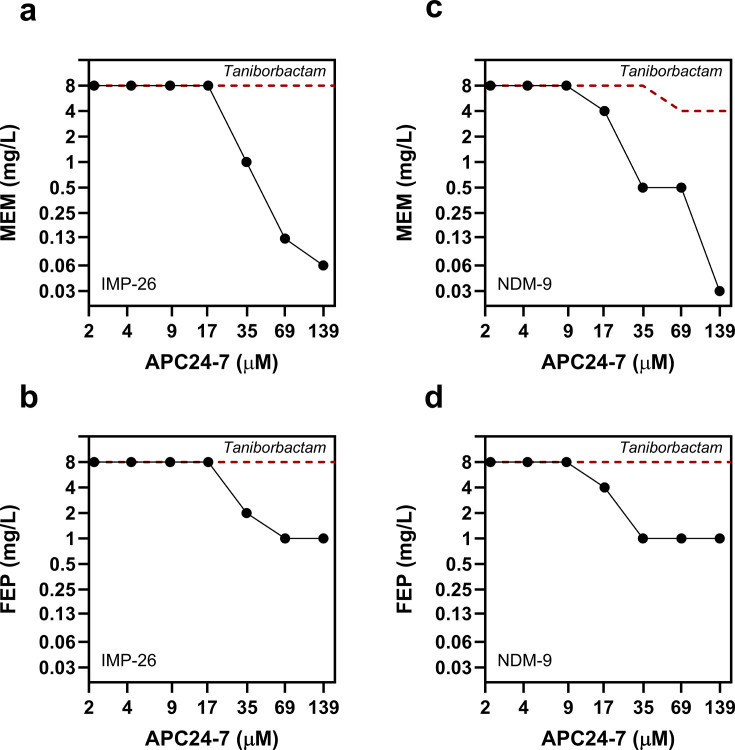
Dose-response curves for meropenem (MEM) and cefepime (FEP) in combination with inhibitors APC24-7 or taniborbactam in isogenic *E. coli* strains. (**a**) MEM/BLI (MP 30-58; IMP-26), (**b**) FEP/BLI (MP 30-58; IMP-26), (**c**) MEM/BLI (MP 30-15; NDM-9), and (**d**) FEP/BLI (MP 30-15; NDM-9). MICs are presented in mg/L, with data points representing observed responses (solid lines) for APC24-7 combinations. The responses in combination with APC24-7 are shown as solid lines. The red dashed lines show the respective MEM- and FEP MICs in combination with taniborbactam. For MIC values, see Tables S6 and S7.

Given the strong concentration-dependent inhibition observed for APC24-7 (Table S4), we conducted checkerboard assays for APC24-7 in combination with either meropenem or cefepime and compared the results to those obtained with taniborbactam. Using isogenic *E. coli* strains producing the taniborbactam-escape variants IMP-26 and NDM-9, we aimed to determine the concentration range required for effective sensitization of these MBL-producing strains. Even at the highest tested concentration (139 µM), taniborbactam did not achieve an efficient reduction (>2-fold) in the MICs of either meropenem or cefepime for the tested isogenic strains (Fig. 2a through d). These findings align with previous studies indicating low-level inhibition by taniborbactam of certain members of the IMP and NDM enzyme families (25, 32, 33). In contrast, APC24-7 in combination with either meropenem or cefepime demonstrated a strong concentration-dependent MIC reduction at concentrations ≥35 µM against isogenic strains producing IMP-26 (Fig. 2a and b) and NDM-9 (Fig. 2c and d). These effects were particularly pronounced for the meropenem/APC24-7 combination, for which the highest tested APC24-7 concentration (139 µM) completely sensitized both the IMP-26- and NDM-9-producing isogenic strains, achieving meropenem MICs of 0.06 mg/L and 0.03 mg/L, respectively (Fig. 2a and c).

### APC24-7 inhibition of NDM-9 and IMP-26 is attenuated in the presence of exogenous Zn^2+^

**Fig 3:**
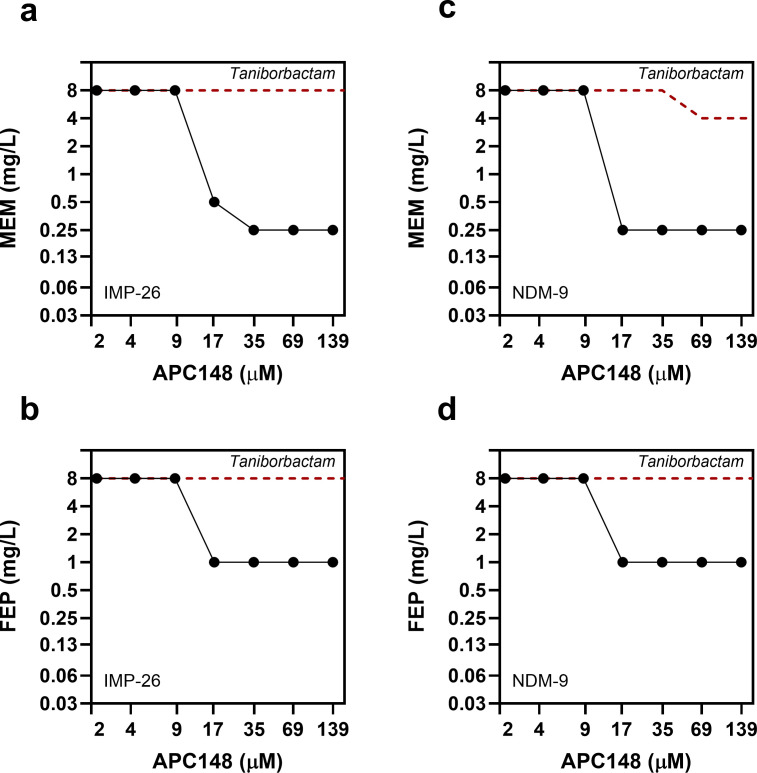
Dose-response curves for meropenem (MEM) or cefepime (FEP) in combination with inhibitors APC148 or taniborbactam in isogenic *E. coli* strains. (**a**) MEM/BLI (MP 30-58; IMP-26), (**b**) FEP/BLI (MP 30-58; IMP-26), (**c**) MEM/BLI (MP 30-15; NDM-9), and (**d**) FEP/BLI (MP 30-15; NDM-9). MICs are presented in mg/L, with data points representing observed responses. The responses in combination with APC148 are shown as solid lines. The red dashed lines show the respective MEM- and FEP MICs in combination with taniborbactam. For MIC values, see Tables S6 and S7.

Although APC24-7 and taniborbactam share a common boronic acid moiety, taniborbactam, even at the highest tested inhibitor concentration (139 µM), demonstrated no substantial resensitization effect against isogenic *E. coli* strains producing either NDM-9 or IMP-26 (Fig. 2a through d). These results suggest that APC24-7’s broadened ability to reverse resistance mediated by these enzymes may be attributed to its chelating moiety. To further investigate the importance of chelation during susceptibility testing, we set up a similar experiment and analyzed the previously described TPA-based chelator APC148 (40). At equimolar concentrations (2.2–139 μM), APC148 in combination with either meropenem or cefepime demonstrated a potent resensitization effect (>16-fold reduction) at concentrations ≥17 µM (Fig. 3a through d) for both IMP-26- and NDM-9-producing *E. coli* strains. Thus, both chelator-containing compounds, APC24-7 and APC148, were able to reverse meropenem and cefepime resistance mediated by these strains. The combined results from checkerboard assays with APC24-7, APC148, and taniborbactam (Fig. 2 and 3) highlight the potential significance of a chelating moiety in sensitizing isogenic *E. coli* strains producing IMP-26 or NDM-9 to meropenem and cefepime.

**Fig 4:**
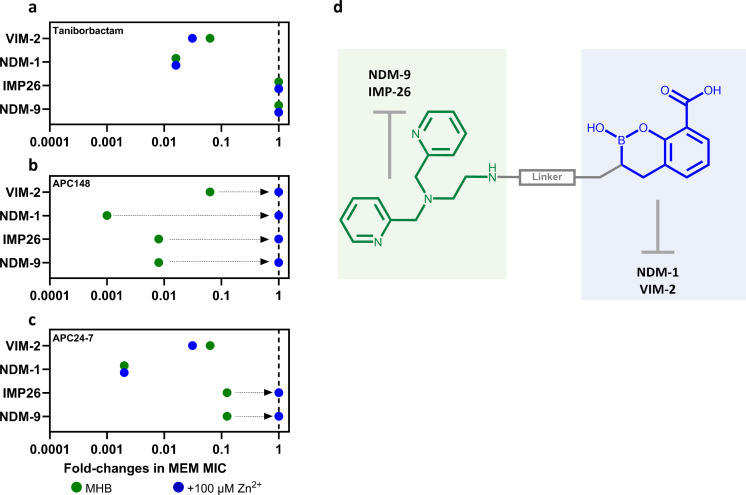
Zn²^+^-dependent inhibitor effect on meropenem susceptibilities. The fold change in meropenem (MEM) MICs is shown for combinations with (**a**) taniborbactam, (**b**) APC148 and (**c**) APC24-7, in Mueller-Hinton broth (MHB) alone and in MHB supplemented with 100 µM Zn²^+^ for isogenic *E. coli* strains producing VIM-2 (MP 30-57), NDM-1 (MP 30-63), IMP-26 (MP 30-58), or NDM-9 (MP 30-15). The results are normalized to MICs without inhibitors and presented on a log10 scale. (**d**) The proposed contributions of APC24-7's structural moieties to its BLI activity are illustrated. For MIC values, see Table S8.

To understand whether APC24-7’s ability to reverse IMP-26- and NDM-9-mediated meropenem resistance was due to the presence of the chelator moiety, we repeated the MIC assays for the isogenic *E. coli* strains in the presence of 100 µM Zn²^+^ (Fig. 4). We hypothesized that an excess of exogenous Zn²^+^ would saturate the chelator, removing its resensitization effect against MBLs. Control strains producing VIM-2 and NDM-1 were included, as taniborbactam’s boronic acid moiety effectively inhibits these MBLs, and Zn²^+^ supplementation should therefore not affect susceptibility due to its transition-state mimicry. As expected, the addition of Zn²^+^ in the taniborbactam assays did not impact the meropenem MICs for NDM-1- or VIM-2-producing strains, consistent with taniborbactam’s mode of action as a transition state analog (Fig. 4a) (21, 57). Furthermore, taniborbactam showed no resensitization effect in strains producing IMP-26 or NDM-9 under either Zn²^+^ condition. In contrast, meropenem/APC148 assays supplemented with Zn²^+^ revealed a restoration of meropenem resistance regardless of the MBL produced, mirroring conditions without inhibitor and suggesting saturation of the chelator moiety (Fig. 4b). In the case of APC24-7 (Fig. 4c), the presence and absence of exogenous Zn²^+^ for the NDM-1- and VIM-2-producing strains yielded similar meropenem MICs (<2-fold). For the strains producing IMP-26 and NDM-9, Zn²^+^ supplementation nullified the resensitization effect of APC24-7, restoring meropenem resistance (Fig. 4d), similarly to APC148.

Thus, APC24-7 represents a hybrid inhibitor with a proposed dual, complementary mode of inhibition. Despite the presumed Zn²^+^-binding properties of APC24-7, meropenem MICs for NDM-1- and VIM-2-producing strains were unaffected by Zn²^+^ supplementation (Fig. 4c), indicating that the boronic acid moiety primarily drives resensitization of strains carrying these MBLs. On the contrary, the reversion of the meropenem resistance of the IMP-26- and NDM-9-producing strains is likely mediated by the presence of the DPED moiety in APC24-7.

## DISCUSSION

While taniborbactam has shown efficacy against MBLs such as NDM-1 and VIM-2, its inhibitory activity against several IMP-type and certain NDM-type enzymes (e.g., IMP-26 and NDM-9) is minimal (Fig. 2 and 3) (33, 34). Several studies have reported a globally increasing prevalence of these enzymes in clinical isolates, potentially hampering the clinical impact of taniborbactam (30, 58–61). In this study, we investigated the SBL-MBL inhibitor APC24-7, which combines a boronic acid structure with a DPED-type chelator to expand its ability to reverse resistance mediated by MBLs that are capable of evading the effect of the boronic acid moiety (Fig. 1). APC24-7 represents a unique case of BLI design, as it combines two distinct moieties with complementary modes of action in the same molecule. The boronic acid moiety of APC24-7 can act on SBL and MBL variants typically inhibited by taniborbactam (22, 25, 26). On the contrary, the DPED-type chelator part likely affects the dependence of MBL enzymes on Zn²^+^, which enables its effect on taniborbactam-escape variants, such as IMP-26 and NDM-9 (Fig. 1 and 4) (38, 39, 42). *In vitro* enzyme kinetics from previous studies suggest that taniborbactam exhibits lower binding affinity (*K*_i_) and/or inhibitor activity (IC_50_) against such escape variants (e.g., IMP-1, IMP-4, IMP-5, NDM-9, and NDM-30), which may be related to the reduced efficacy of taniborbactam toward isolates carrying these enzyme variants (21, 32). The complementary character of APC24-7 likely overcomes this limitation by leveraging its DPED-type chelating moiety to reverse resistance mediated by such escape variants.

 BL/BLI combinations, such as ceftazidime/avibactam and meropenem/vaborbactam, have significantly enhanced our ability to treat infections caused by SBL-producing pathogens (3, 13–16). However, the co-expression of SBLs and MBLs in many clinical isolates substantially limits our current treatment options (17–20). One of the few available alternatives is the recently approved combination aztreonam/avibactam (2024) for infections caused by MBL-producing pathogens (35, 62–64). Despite aztreonam's inherent resilience to MBL-mediated hydrolysis, concerns remain regarding the co-expression of SBLs capable of hydrolyzing aztreonam and target modifications, such as in PBP-3, leading to decreased aztreonam susceptibility (35, 65–69). These factors could compromise the synergy of aztreonam/avibactam, potentially affecting its clinical viability. This highlights the dire need for a complement of alternative drug development strategies with the ability to target a broad range of β-lactamases across all Ambler classes and particularly class B enzymes.

While various approaches to MBL inhibitor development are under investigation, the reliance of these enzymes on Zn²^+^ for their hydrolytic activity has made chelators an appealing focus for drug development (42, 70, 71). While non-selective (Ca-EDTA) Zn^2+^ chelation strategies have been employed in murine models without apparent toxicity, the development of chelator-based inhibitors without significant off-target effects remains a considerable challenge (31, 71–74). Alternatively, structurally related compounds to AP24-7, such as APC148, have demonstrated minimal inhibition of the human glyoxylase II, but measurable effects on other human enzymes such as matrix metalloproteinase-14 (MMP-14) (40, 43). Yet, APC148 did not cause acute toxicity in mice, and a SAD phase 1 clinical study (NCT06360640) has been completed. Nonetheless, future studies evaluating potential safety, off-target effects, and the biochemical mode of inhibition of APC24-7 are necessary to answer questions on its selectivity and specificity.

Taken together, we have investigated APC24-7, which exemplifies how combining complementary inhibitor motifs, such as chelators and boronic acid transition state analogs, can design hybrid SBL and MBL inhibitors with a broadened ability to reverse MBL- and SBL-mediated resistance (Fig. 1 to 3). This strategy is not limited to the DPED-type chelator- and boronic acid motifs tested here, but represents a promising design tool for the development of inhibitors with complementary modes of action.

## MATERIALS AND METHODS

### Synthesis of APC24-7 and APC148

The synthesis of APC24-7 is described in the Protocol S1. The synthesis of APC148 (previously ZN148) has been described previously (40). NMR spectroscopy was performed using a Bruker Avance AVIIIHD400 spectrometer operating at 400 MHz (^1^H NMR) and 101 MHz (^13^C NMR). All spectra were recorded at room temperature and have been referenced relative to the residual solvent signal. Molecular masses were verified using electrospray ionization-mass spectrometry (Bruker maXis II ETD spectrometer and an Agilent 1100 HPLC-MS instrument).

### Chemical compounds, media, and strains

Amoxicillin (Merck, Darmstadt, Germany) and aztreonam (Merck, Darmstadt, Germany) were dissolved in dimethyl sulfoxide (DMSO). Ceftazidime (Merck, Darmstadt, Germany) and cefepime (Merck, Darmstadt, Germany) were dissolved in phosphate-buffered saline (PBS) solution (Gibco, pH 7.4). Premade Sensititre plates set with meropenem (TREK Diagnostic Systems/Thermo Fisher Scientific, East Grinstead, United Kingdom) were used to determine the MIC of meropenem. The dinatrium salt of compound APC24-7 (Kappa Solutions, Oslo, Norway) was dissolved in diethyl pyrocarbonate-treated water (Invitrogen) to a stock concentration of 100 mM (56.5 g/L). Taniborbactam (MedChem Express) was dissolved in DMSO to a stock concentration of 14 mM (6.4 g/L). Meropenem (Merck) was dissolved in dH_2_O and ampicillin (Sigma Aldrich) in dH_2_O. Chloramphenicol was dissolved in 70% ethanol to a stock of 25 mg/mL. Luria-Bertani (LB) broth was prepared in-house by mixing 10 g /L Tryptone (Thermo Fisher), 10 g/L NaCl (Thermo Fisher), and 5 g/L yeast extract (Thermo Fisher) in dH_2_O. Strains used and constructed in this study are summarized in Table S1. Primers constructed and used were purchased from Integrated DNA Technologies and displayed in Table S9.

### Susceptibility testing

MICs for clinical isolates were determined in a broth microdilution assay according to Clinical and Laboratory Standards Institute (CLSI) (75), using cation-adjusted Mueller-Hinton broth (Thermo Fisher Scientific, Lenexa, USA). The direct colony suspension method was utilized by preparing an inoculum of an OD_600_ 0.17, except for *E. coli* #36 in which 0.10 was used, which equated to a concentration of 10^8^ colony-forming units (CFU) per mL. Inoculum controls were included for all assays (76).

MICs for all clinical isolates were determined alone and in combination with inhibitors for all BLs tested, either using manually prepared plates of aztreonam, cefepime, ceftazidime, and amoxicillin or premade plates of meropenem (Sensititre), and all antibiotics tested at a twofold increasing concentration of 0.03–64 mg/L (Table 1; Table S3). Compound APC24-7 was tested at a fixed concentration of 14, 28, and 57 µM (8, 16, or 32 mg/L) with meropenem, and for all other BLs tested at the highest inhibitor concentration to be able to compare the efficiency of the BI/BLI combinations. Taniborbactam was tested at a fixed equimolar concentration (6.5, 13, and 26 mg/L) and only evaluated with meropenem. A minimum of 2–3 replicates was performed for all combinations. Assay plates were incubated at 37°C without shaking for 16–20 h, and the results were visually interpreted in accordance with EUCAST guidelines and clinical breakpoint table version 15.0 (www.eucast.org).

Susceptibility testing of isogenic *E. coli* E. cloni 10G (Table 2) was determined by both the microdilution assay. APC247, APC148, and taniborbactam were tested alone toward isogenic *E. coli* without vector (21-05) as well as WT with empty pUN vector (MP32-64) to control for any antimicrobial activity in a twofold increasing concentration of BLI to a maximum of 906 µM (512, 464, and 419 mg/L, respectively) (Table S2). Overnight cultures were prepared in LB supplemented with 25 mg/L chloramphenicol at 37°C under shaking at 700 rpm. The overnight cultures were diluted in PBS to 10^6^ CFU/ mL, followed by inoculation in LB to ~10^5^ CFU in 384-well plates (VWR, Radnor, PA, USA). Plates were prepared with twofold increasing concentrations of meropenem and cefepime (0.004–64 mg/L) in combination with APC24-7 or taniborbactam fixed at 9 µM (4.9 and 4.0 mg/L, respectively) to a total inoculated volume of 100 µL Plates were incubated at 37°C without shaking for 19–21 h. OD_600_ was determined in a SpectraMax Plus plate reader (Molecular Devices). In this setup, the MIC was defined as the first well with an optical density comparable to the negative control and determined based on at least biological triplicates.

MIC assays in the presence and absence of exogenous Zn^2+^ were performed in cation-adjusted Mueller-Hinton broth supplemented with 100 µM ZnSO_4_ (Sigma-Aldrich) in premade Sensititre plates. The concentrations of APC148, APC24-7, and taniborbactam were fixed (respectively) at 18, 20, and 16 mg/L (35 µM) for NDM-1, NDM-9, and VIM-2 and 36, 40, and 32 mg/L (70 µM) for IMP-26. Final inoculum was 10^5^ CFU/mL and plates were incubated at 37°C for 16–20 h, and results were visually interpreted as the lowest concentration that resulted in no visible growth.

The potential antimicrobial effect of APC24-7 was assessed by incubating a selection of the strains in a twofold increasing concentration to a maximum of 906 µM (512 mg/L) (Table S2).

### Cloning

For susceptibility testing in isogenic *E. coli* E. cloni 10 G strains, a low-to-medium-copy-number vector (pUN, pA15 origin, ~15 copies/cell) (50, 51) was used, carrying a chloramphenicol resistance marker. The vectors carrying pUN-*bla*_NDM-1_, pUN-*bla*_IMP-26_*,* pUN-*bla*_VIM-2_*,* pUN-*bla*_OXA-48_*,* pUN-*bla*_KPC-2_*,* and pUN-*bla*_CTX-M-15_ were constructed previously (51, 77, 78). NDM variants (using NDM-1 as a template) and OXA-163 (using OXA-48 as a template) were constructed using GoldenGate cloning as described before (Table S1) (51). In brief, templates were amplified with primers shown in Table S9 to construct NDM-variants NDM-4 (primer CF80), NDM-7 (primer CF84), NDM-9 (primer CF86), and NDM-16 (primers CF82 and CF83) as well as SBL OXA-163 (primer CF54) using Phusion polymerase (New England Biolabs) followed by digestion with LguI/DpnI (Thermo Fisher). Ligation was performed using T4 DNA ligase (Thermo Fisher), and the ligated product was transformed into *E. coli* E. cloni 10G. Cells were recovered at 37°C for 1 h in LB broth and selected on LB agar plates containing 25 mg/L chloramphenicol. Verification of the correct insert was performed by Sanger sequencing (Eurofins, Germany) using primers CF7 and CF8 (Table S9).

For enzyme expression and purification, NDM-9 (amino acid 29Gly to 270Arg) was subcloned without the signal peptide from *E. coli* MP 30-15 into expression vector pDEST-17 (pOML-213) (79). Vector backbone and insert were PCR amplified using Phusion polymerase (New England Biolabs) and primers P3/P4 and P5/P6, respectively (Table S9). This resulted in NDM-9 harboring a 6-His-maltose-binding protein (MBP) tag at the N-terminal followed by a TEV-cleaving site (79). Amplicons were digested by *DpnI* (New England Biolabs), and Gibson recombination (New England Biolabs) was performed according to the manufacturer’s protocol. Gibson product was transformed into *E. coli* BL21 (DE3), and transformants were selected on LB agar containing 100 mg/L ampicillin. The insert was verified by Sanger sequencing using primers P1/P2.

### Expression and purification of NDM-9

Protein expression and purification were performed as previously described (79–81). In short, cells were grown in LB supplemented with 100 µM Zn^2+^(ZnSO_4_, Sigma-Aldrich) to an OD_600_ ~0.5, and protein expression was induced with 1 mM isopropyl-β-D-thiogalactopyranoside. Cultures were shaken at 225 rpm and incubated for 16–20 h at 15°C. The cell pellet was resuspended in 50 mM HEPES (Merck), pH 7.5 supplemented with 100 µM Zn^2+^ (buffer A) and proteinase inhibitor (Sigma Aldrich, 1×). The cell lysate was purified on amylose resin (New England Biolabs), and the enzyme was eluted using buffer A supplemented with 20 mM maltose (Sigma-Aldrich). The size and purity of the protein were assessed by SDS-PAGE (Bio-Rad, USA). NDM-1 (81), VIM-2 (82), VIM-7 (83), SHD-1, ECV-1, MYO-1 (48), and OXA-48 (80) were previously purified.

### IC**_50_** determination of APC24-7

IC_50_ determination with purified enzymes was performed as previously described (40). In brief, enzymes were diluted in 50 mM HEPES, pH 7.5, supplemented with 1 µM Zn^2+^. For OXA-48, 50 mM HEPES, pH 7.5, was supplemented with 50 mM sodium bicarbonate buffer solution (Sigma Aldrich). Final enzyme concentrations are shown in Table S5. Enzymes and inhibitors were mixed at various inhibitor concentrations, and absorbance was measured by spectrophotometry (Table S5). For NDM-1 and MBP-NDM-9, the hydrolysis of meropenem (24 µM) was monitored at 300 nm using UV- transparent 96-well plates (Corning, Kennebunk, ME). For all other enzymes, nitrocefin (24 µM, Millipore Sigma, USA) hydrolysis was monitored at 480 nm. The residual enzyme activity at various inhibitor concentrations was calculated in comparison to the initial reaction velocity of the inhibitor-free and enzyme-free control. Dose-response curves were fitted using nonlinear regression in GraphPad Prism v. 10.5.0. All tests were performed at least in duplicates at 25°C.

### Checkerboard assays

Overnight cultures of isogenic *E. coli* producing either NDM-9 or IMP-26 were prepared in LB broth supplemented with 25 mg/L chloramphenicol and incubated at 37°C shaking at 220 rpm. Overnight cultures were diluted 1:1,000 in LB broth to a final concentration of 10^5^ CFU in the 96-well assay plate. Both inhibitor and antibiotic were prepared by a twofold dilution series and added to a 96-well assay plate in accordance with a standard checkerboard setup, including a growth control (no BL or BLI). The meropenem and cefepime concentration ranges were 0.008–8 mg/L, and all BLIs tested at 2.2–139 µM for taniborbactam, APC24-7, and APC148 (maximum concentration of 64, 78, and 71 mg/L, respectively). Plates were incubated at 37°C without shaking for 20 h, and OD_600_ values were determined in a SpectraMax Plus plate reader and MICs at different inhibitor concentrations were determined, based on a minimum of two biological replicates.

## SUPPLEMENTAL MATERIAL

Supplemental material msphere.00418-25-s0001.docx Protocol S1, Fig. S1 and S2, and Tables S1-S9.

ASM does not own the copyrights to Supplemental Material that may be linked to, or accessed through, an article. The authors have granted ASM a non-exclusive, world-wide license to publish the Supplemental Material files. Please contact the corresponding author directly for reuse.

## References

[1] Collaborators GBDAR. 2024. Global burden of bacterial antimicrobial resistance 1990–2021: a systematic analysis with forecasts to 2050. Lancet 404:1199–1226. doi:10.1016/S0140-6736(24)01867-1 39299261 PMC11718157

[2] Ambler RP. 1980. The structure of beta-lactamases. Philos Trans R Soc Lond B Biol Sci 289:321–331. doi:10.1098/rstb.1980.0049 6109327

[3] Tooke CL, Hinchliffe P, Bragginton EC, Colenso CK, Hirvonen VHA, Takebayashi Y, Spencer J. 2019. β-lactamases and β-lactamase inhibitors in the 21st century. J Mol Biol 431:3472–3500. doi:10.1016/j.jmb.2019.04.002 30959050 PMC6723624

[4] Salahuddin P, Kumar A, Khan AU. 2018. Structure, function of serine and metallo-β-lactamases and their inhibitors. Curr Protein Pept Sci 19:130–144. doi:10.2174/0929866524666170724160623 28745223

[5] Garau G, Di Guilmi AM, Hall BG. 2005. Structure-based phylogeny of the metallo-beta-lactamases. Antimicrob Agents Chemother 49:2778–2784. doi:10.1128/AAC.49.7.2778-2784.2005 15980349 PMC1168685

[6] Bush K. 2023. Classification for β-lactamases: historical perspectives. Expert Rev Anti Infect Ther 21:513–522. doi:10.1080/14787210.2023.2194633 36951174

[7] Bebrone C. 2007. Metallo-beta-lactamases (classification, activity, genetic organization, structure, zinc coordination) and their superfamily. Biochem Pharmacol 74:1686–1701. doi:10.1016/j.bcp.2007.05.021 17597585

[8] Shields RK, Doi Y. 2020. Aztreonam combination therapy: an answer to metallo-β-lactamase-producing gram-negative bacteria? Clin Infect Dis 71:1099–1101. doi:10.1093/cid/ciz1159 31802110 PMC7428391

[9] Ling Z, Farley AJM, Lankapalli A, Zhang Y, Premchand-Branker S, Cook K, Baran A, Gray-Hammerton C, Orbegozo Rubio C, Suna E, Mathias J, Brem J, Sands K, Nieto-Rosado M, Trush MM, Rakhi NN, Martins W, Zhou Y, Schofield CJ, Walsh T. 2024. The triple combination of meropenem, avibactam, and a metallo-β-lactamase inhibitor optimizes antibacterial coverage against different β-lactamase producers. Engineering (Beijing) 38:124–132. doi:10.1016/j.eng.2024.02.010 40109291 PMC11913740

[10] Tan X, Kim HS, Baugh K, Huang Y, Kadiyala N, Wences M, Singh N, Wenzler E, Bulman ZP. 2021. Therapeutic options for metallo-β-lactamase-producing Enterobacterales. Infect Drug Resist 14:125–142. doi:10.2147/IDR.S246174 33500635 PMC7822077

[11] Bush K, Fisher JF. 2011. Epidemiological expansion, structural studies, and clinical challenges of new β-lactamases from gram-negative bacteria. Annu Rev Microbiol 65:455–478. doi:10.1146/annurev-micro-090110-102911 21740228

[12] Zhang S, Liao X, Ding T, Ahn J. 2024. Role of β-lactamase inhibitors as potentiators in antimicrobial chemotherapy targeting gram-negative bacteria. Antibiotics (Basel) 13:260. doi:10.3390/antibiotics13030260 38534695 PMC10967447

[13] Bush K. 2018. Game changers: new β-lactamase inhibitor combinations targeting antibiotic resistance in gram-negative bacteria. ACS Infect Dis 4:84–87. doi:10.1021/acsinfecdis.7b00243 29232103

[14] Carmeli Y, Armstrong J, Laud PJ, Newell P, Stone G, Wardman A, Gasink LB. 2016. Ceftazidime-avibactam or best available therapy in patients with ceftazidime-resistant Enterobacteriaceae and Pseudomonas aeruginosa complicated urinary tract infections or complicated intra-abdominal infections (REPRISE): a randomised, pathogen-directed, phase 3 study. Lancet Infect Dis 16:661–673. doi:10.1016/S1473-3099(16)30004-4 27107460

[15] de Jonge BLM, Karlowsky JA, Kazmierczak KM, Biedenbach DJ, Sahm DF, Nichols WW. 2016. In vitro susceptibility to ceftazidime-avibactam of carbapenem-nonsusceptible Enterobacteriaceae isolates collected during the INFORM global surveillance study (2012 to 2014). Antimicrob Agents Chemother 60:3163–3169. doi:10.1128/AAC.03042-15 26926648 PMC4862516

[16] Kang SJ, Kim DH, Lee BJ. 2024. Metallo-β-lactamase inhibitors: a continuing challenge for combating antibiotic resistance. Biophys Chem 309:107228. doi:10.1016/j.bpc.2024.107228 38552402

[17] Medeiros AA. 1997. Evolution and dissemination of beta-lactamases accelerated by generations of beta-lactam antibiotics. Clin Infect Dis 24 Suppl 1:S19–45. doi:10.1093/clinids/24.supplement_1.s19 8994778

[18] Salvia T, Dolma KG, Dhakal OP, Khandelwal B, Singh LS. 2022. Phenotypic detection of ESBL, AmpC, MBL, and their co-occurrence among MDR Enterobacteriaceae isolates. J Lab Physicians 14:329–335. doi:10.1055/s-0042-1744239 36119416 PMC9473942

[19] Marshall S, Hujer AM, Rojas LJ, Papp-Wallace KM, Humphries RM, Spellberg B, Hujer KM, Marshall EK, Rudin SD, Perez F, Wilson BM, Wasserman RB, et al.. 2017. Can ceftazidime-avibactam and aztreonam overcome β-lactam resistance conferred by metallo-β-lactamases in Enterobacteriaceae? Antimicrob Agents Chemother 61:e02243-16. doi:10.1128/AAC.02243-16 28167541 PMC5365724

[20] Rajer F, Allander L, Karlsson PA, Sandegren L. 2022. Evolutionary trajectories toward high-level β-lactam/β-lactamase inhibitor resistance in the presence of multiple β-lactamases. Antimicrob Agents Chemother 66:e00290-22. doi:10.1128/aac.00290-22 35652643 PMC9211440

[21] Hamrick JC, Docquier J-D, Uehara T, Myers CL, Six DA, Chatwin CL, John KJ, Vernacchio SF, Cusick SM, Trout REL, Pozzi C, De Luca F, et al.. 2020. VNRX-5133 (taniborbactam), a broad-spectrum inhibitor of serine- and metallo-β-lactamases, restores activity of cefepime in Enterobacterales and Pseudomonas aeruginosa. Antimicrob Agents Chemother 64:e01963-19. doi:10.1128/AAC.01963-19 31871094 PMC7038240

[22] Liu B, Trout REL, Chu G-H, McGarry D, Jackson RW, Hamrick JC, Daigle DM, Cusick SM, Pozzi C, De Luca F, Benvenuti M, Mangani S, Docquier J-D, Weiss WJ, Pevear DC, Xerri L, Burns CJ. 2020. Discovery of taniborbactam (VNRX-5133): a broad-spectrum serine- and metallo-β-lactamase inhibitor for carbapenem-resistant bacterial infections. J Med Chem 63:2789–2801. doi:10.1021/acs.jmedchem.9b01518 31765155 PMC7104248

[23] Krajnc A, Brem J, Hinchliffe P, Calvopiña K, Panduwawala TD, Lang PA, Kamps JJAG, Tyrrell JM, Widlake E, Saward BG, Walsh TR, Spencer J, Schofield CJ. 2019. Bicyclic boronate VNRX-5133 inhibits metallo- and serine-β-lactamases. J Med Chem 62:8544–8556. doi:10.1021/acs.jmedchem.9b00911 31454231 PMC6767355

[24] Moeck G, Gasink LB, Mendes RE, Woosley LN, Dorr M, Chen H, Wagenlehner FM, Henkel T, McGovern PC. 2024. Patient outcomes by baseline pathogen resistance phenotype and genotype in CERTAIN-1, a phase 3 study of cefepime-taniborbactam versus meropenem in adults with complicated urinary tract infection. Antimicrob Agents Chemother 68:e00236-24. doi:10.1128/aac.00236-24 38780262 PMC11232400

[25] Mushtaq S, Vickers A, Doumith M, Ellington MJ, Woodford N, Livermore DM. 2021. Activity of β-lactam/taniborbactam (VNRX-5133) combinations against carbapenem-resistant gram-negative bacteria. J Antimicrob Chemother 76:160–170. doi:10.1093/jac/dkaa391 33305800

[26] Karlowsky JA, Hackel MA, Wise MG, Six DA, Uehara T, Daigle DM, Cusick SM, Pevear DC, Moeck G, Sahm DF. 2023. In vitro activity of cefepime-taniborbactam and comparators against clinical isolates of gram-negative bacilli from 2018 to 2020: results from the Global Evaluation of Antimicrobial Resistance via Surveillance (GEARS) program. Antimicrob Agents Chemother 67:e01281-22. doi:10.1128/aac.01281-22 36541767 PMC9872668

[27] Lasko MJ, Nicolau DP, Asempa TE. 2022. Clinical exposure–response relationship of cefepime/taniborbactam against gram-negative organisms in the murine complicated urinary tract infection model. J Antimicrob Chemother 77:443–447. doi:10.1093/jac/dkab405 34747449 PMC8809191

[28] Hernández-García M, García-Castillo M, Ruiz-Garbajosa P, Bou G, Siller-Ruiz M, Pitart C, Gracia-Ahufinger I, Mulet X, Pascual Á, Tormo N, Cantón R. 2022. In vitro activity of cefepime-taniborbactam against carbapenemase-producing Enterobacterales and Pseudomonas aeruginosa isolates recovered in Spain. Antimicrob Agents Chemother 66:e02161-21. doi:10.1128/aac.02161-21 35007130 PMC8923209

[29] Roach EJ, Uehara T, Daigle DM, Six DA, Khursigara CM. 2021. The next-generation β-lactamase inhibitor taniborbactam restores the morphological effects of cefepime in KPC-producing Escherichia coli. Microbiol Spectr 9:e00918-21. doi:10.1128/Spectrum.00918-21 34494877 PMC8557880

[30] Pongchaikul P, Mongkolsuk P. 2022. Comprehensive analysis of imipenemase (IMP)-type metallo-β-lactamase: a global distribution threatening Asia. Antibiotics (Basel) 11:236. doi:10.3390/antibiotics11020236 35203838 PMC8868347

[31] Mojica MF, Rossi MA, Vila AJ, Bonomo RA. 2022. The urgent need for metallo-β-lactamase inhibitors: an unattended global threat. Lancet Infect Dis 22:e28–e34. doi:10.1016/S1473-3099(20)30868-9 34246322 PMC8266270

[32] Le Terrier C, Freire S, Viguier C, Findlay J, Nordmann P, Poirel L. 2024. Relative inhibitory activities of the broad-spectrum β-lactamase inhibitor xeruborbactam in comparison with taniborbactam against metallo-β-lactamases produced in Escherichia coli and Pseudomonas aeruginosa Antimicrob Agents Chemother 68:e01570-23. doi:10.1128/aac.01570-23 38727224 PMC11620488

[33] Le Terrier C, Gruenig V, Fournier C, Nordmann P, Poirel L. 2023. NDM-9 resistance to taniborbactam. Lancet Infect Dis 23:401–402. doi:10.1016/S1473-3099(23)00069-5 36796395

[34] Tamma PD, Munita JM. 2024. The metallo-β-lactamases strike back: emergence of taniborbactam escape variants. Antimicrob Agents Chemother 68:e01510-23. doi:10.1128/aac.01510-23 38174925 PMC10848767

[35] Grabein B, Arhin FF, Daikos GL, Moore LSP, Balaji V, Baillon-Plot N. 2024. Navigating the current treatment landscape of metallo-β-lactamase-producing gram-negative infections: what are the limitations? Infect Dis Ther 13:2423–2447. doi:10.1007/s40121-024-01044-8 39352652 PMC11499561

[36] Huang Z, Zhang X, Bosch M, Smith SJ, Lippard SJ. 2013. Tris(2-pyridylmethyl)amine (TPA) as a membrane-permeable chelator for interception of biological mobile zinc. Metallomics 5:648–655. doi:10.1039/c3mt00103b 23715510 PMC3730853

[37] Schnaars C, Kildahl-Andersen G, Prandina A, Popal R, Radix S, Le Borgne M, Gjøen T, Andresen AMS, Heikal A, Økstad OA, Fröhlich C, Samuelsen Ø, et al.. 2018. Synthesis and preclinical evaluation of TPA-based zinc chelators as metallo-β-lactamase inhibitors. ACS Infect Dis 4:1407–1422. doi:10.1021/acsinfecdis.8b00137 30022668

[38] Kildahl-Andersen G, Schnaars C, Prandina A, Radix S, Le Borgne M, Jordheim LP, Gjøen T, Andresen AMS, Lauksund S, Fröhlich C, Samuelsen Ø, Rongved P, Åstrand OAH. 2019. Synthesis and biological evaluation of zinc chelating compounds as metallo-β-lactamase inhibitors. Medchemcomm 10:528–537. doi:10.1039/c8md00578h 31057732 PMC6482411

[39] Prandina A, Radix S, Le Borgne M, Jordheim LP, Bousfiha Z, Fröhlich C, Leiros H-K, Samuelsen Ø, Frøvold E, Rongved P, Åstrand OAH. 2019. Synthesis and biological evaluation of new dipicolylamine zinc chelators as metallo-β-lactamase inhibitors. Tetrahedron 75:1525–1540. doi:10.1016/j.tet.2019.02.004

[40] Samuelsen Ø, Åstrand OAH, Fröhlich C, Heikal A, Skagseth S, Carlsen TJO, Leiros H-K, Bayer A, Schnaars C, Kildahl-Andersen G, Lauksund S, Finke S, Huber S, et al.. 2020. ZN148 is a modular synthetic metallo-β-lactamase inhibitor that reverses carbapenem resistance in gram-negative pathogens in vivo. Antimicrob Agents Chemother 64:e02415-19. doi:10.1128/AAC.02415-19 32179522 PMC7269481

[41] Siemann S, Brewer D, Clarke AJ, Dmitrienko GI, Lajoie G, Viswanatha T. 2002. IMP-1 metallo-beta-lactamase: effect of chelators and assessment of metal requirement by electrospray mass spectrometry. Biochim Biophys Acta 1571:190–200. doi:10.1016/s0304-4165(02)00258-1 12090933

[42] Azumah R, Dutta J, Somboro AM, Ramtahal M, Chonco L, Parboosing R, Bester LA, Kruger HG, Naicker T, Essack SY, Govender T. 2016. In vitro evaluation of metal chelators as potential metallo- β -lactamase inhibitors. J Appl Microbiol 120:860–867. doi:10.1111/jam.13085 26849010

[43] Rahman F, Wushur I, Malla N, Åstrand OAH, Rongved P, Winberg J-O, Sylte I. 2022. Zinc-chelating compounds as inhibitors of human and bacterial zinc metalloproteases. Molecules 27:56. doi:10.3390/molecules27010056 PMC8746695 35011288

[44] Brem J, Cain R, Cahill S, McDonough MA, Clifton IJ, Jiménez-Castellanos J-C, Avison MB, Spencer J, Fishwick CWG, Schofield CJ. 2016. Structural basis of metallo-β-lactamase, serine-β-lactamase and penicillin-binding protein inhibition by cyclic boronates. Nat Commun 7:12406. doi:10.1038/ncomms12406 27499424 PMC4979060

[45] Castanheira M, Doyle TB, Collingsworth TD, Sader HS, Mendes RE. 2021. Increasing frequency of OXA-48-producing Enterobacterales worldwide and activity of ceftazidime/avibactam, meropenem/vaborbactam and comparators against these isolates. J Antimicrob Chemother 76:3125–3134. doi:10.1093/jac/dkab306 34459890 PMC8598286

[46] Lee CR, Lee JH, Park KS, Kim YB, Jeong BC, Lee SH. 2016. Global dissemination of carbapenemase-producing Klebsiella pneumoniae: epidemiology, genetic context, treatment options, and detection methods. Front Microbiol 7:895. doi:10.3389/fmicb.2016.00895 27379038 PMC4904035

[47] Galleni M, Lamotte-Brasseur J, Rossolini GM, Spencer J, Dideberg O, Frère JM, Metallo-β-lactamases Working G. 2001. Standard numbering scheme for class B beta-lactamases. Antimicrob Agents Chemother 45:660–663. doi:10.1128/AAC.45.3.660-663.2001 11181339 PMC90352

[48] Fröhlich Christopher, Sørum V, Huber S, Samuelsen Ø, Berglund F, Kristiansson E, Kotsakis SD, Marathe NP, Larsson DGJ, Leiros H-KS. 2020. Structural and biochemical characterization of the environmental MBLs MYO-1, ECV-1 and SHD-1. J Antimicrob Chemother 75:2554–2563. doi:10.1093/jac/dkaa175 32464640 PMC7443720

[49] King DT, Sobhanifar S, Strynadka NCJ. 2017. The mechanisms of resistance to β-lactam antibiotics, p 177–201. In Berghuis A, Matlashewski G, Wainberg MA, Sheppard D, Gotte M (ed), Handbook of antimicrobial resistance. Springer New York, New York, NY.

[50] Fröhlich C, Gama JA, Harms K, Hirvonen VHA, Lund BA, van der Kamp MW, Johnsen PJ, Samuelsen Ø, Leiros H-KS. 2021. Cryptic β-lactamase evolution is driven by low β-lactam concentrations. mSphere 6:e00108-21. doi:10.1128/mSphere.00108-21 33910990 PMC8092134

[51] Fröhlich C, Sørum V, Tokuriki N, Johnsen PJ, Samuelsen Ø. 2022. Evolution of β-lactamase-mediated cefiderocol resistance. J Antimicrob Chemother 77:2429–2436. doi:10.1093/jac/dkac221 35815680 PMC9410664

[52] Dorr MB, Gasink L, Henkel T, Moeck G, Chen H, McConnell SA, McGovern P. 2023. 2513. CERTAIN-1 subgroup analysis: a phase 3 study of cefepime-taniborbactam efficacy in the treatment of complicated urinary tract infections (cUTI). Open Forum Infect Dis 10. doi:10.1093/ofid/ofad500.2131

[53] Moeck G, Gasink L, Dorr MB, Chen H, Woosley L, Mendes RE, Wagenlehner F, Henkel T, McGovern P. 2023. 2523. outcomes by resistance phenotype and genotype among baseline pathogen in patients with complicated urinary tract infection (cUTI) in the phase 3 CERTAIN-1 study. Open Forum Infect Dis 10. doi:10.1093/ofid/ofad500.2141 PMC11232400 38780262

[54] Stojanoski V, Hu L, Sankaran B, Wang F, Tao P, Prasad BVV, Palzkill T. 2021. Mechanistic basis of OXA-48-like β-lactamases’ hydrolysis of carbapenems. ACS Infect Dis 7:445–460. doi:10.1021/acsinfecdis.0c00798 33492952 PMC8571991

[55] Hirvonen VHA, Spencer J, van der Kamp MW. 2021. Antimicrobial resistance conferred by OXA-48 β-lactamases: towards a detailed mechanistic understanding. Antimicrob Agents Chemother 65:e00184-21. doi:10.1128/AAC.00184-21 33753332 PMC8316048

[56] Maryam L, Khan AU. 2017. Structural insight into mode of binding of meropenem to CTX-M-15 type β-lactamase. Int J Biol Macromol 96:78–86. doi:10.1016/j.ijbiomac.2016.12.032 27986632

[57] Drusin SI, Le Terrier C, Poirel L, Bonomo RA, Vila AJ, Moreno DM. 2024. Structural basis of metallo-β-lactamase resistance to taniborbactam. Antimicrob Agents Chemother 68:e0116823. doi:10.1128/aac.01168-23 38063400 PMC10848773

[58] Le Terrier C, Nordmann P, Buchs C, Di DYW, Rossolini GM, Stephan R, Castanheira M, Poirel L. 2023. Wide dissemination of gram-negative bacteria producing the taniborbactam-resistant NDM-9 variant: a one health concern. J Antimicrob Chemother 78:2382–2384. doi:10.1093/jac/dkad210 37394537 PMC10477121

[59] Oueslati S, Emeraud C, Grosperrin V, Levy M, Cotellon G, Creton E, Gauthier L, Bonnin RA, Naas T, Dortet L. 2021. Polyclonal dissemination of NDM-1- and NDM-9-producing Escherichia coli and Klebsiella pneumoniae in french polynesia. Antimicrob Agents Chemother 65:e02437-20. doi:10.1128/AAC.02437-20 33495221 PMC8097482

[60] Kishi R, Nakano R, Nakano A, Harimoto T, Taniguchi R, Ando S, Suzuki Y, Yamaguchi K, Kitagawa D, Horiuchi S, Tsubaki K, Morita R, Kawabe T, Yano H. 2024. Prevalence of carbapenem-resistant Enterobacterales with bla _IMP-6_ predominance in hospitals from 2018 to 2021 in Nara, Japan. JAC Antimicrob Resist 6:dlae135. doi:10.1093/jacamr/dlae135 39165366 PMC11334064

[61] Falcone M, Giordano C, Barnini S, Tiseo G, Leonildi A, Malacarne P, Menichetti F, Carattoli A. 2020. Extremely drug-resistant NDM-9-producing ST147 Klebsiella pneumoniae causing infections in Italy, May 2020. Euro Surveill 25:2001779. doi:10.2807/1560-7917.ES.2020.25.48.2001779 33272354 PMC7716400

[62] Mauri C, Maraolo AE, Di Bella S, Luzzaro F, Principe L. 2021. The revival of aztreonam in combination with avibactam against metallo-β-lactamase-producing gram-negatives: a systematic review of in vitro studies and clinical cases. Antibiotics (Basel) 10:1012. doi:10.3390/antibiotics10081012 34439062 PMC8388901

[63] Bhatnagar A, Boyd S, Sabour S, Bodnar J, Nazarian E, Peinovich N, Wagner C, Craft B, Snippes Vagnone P, Simpson J, Stone VN, Therrien M, et al.. 2021. Aztreonam-avibactam susceptibility testing program for metallo-beta-lactamase-producing Enterobacterales in the antibiotic resistance laboratory network, march 2019 to december 2020. Antimicrob Agents Chemother 65:e0048621. doi:10.1128/AAC.00486-21 34060895 PMC8284474

[64] Emeraud C, Bernabeu S, Dortet L. 2023. In vitro susceptibility of aztreonam-vaborbactam, aztreonam-relebactam and aztreonam-avibactam associations against metallo-β-lactamase-producing gram-negative bacteria. Antibiotics (Basel) 12:1493. doi:10.3390/antibiotics12101493 37887194 PMC10604182

[65] Alm RA, Johnstone MR, Lahiri SD. 2015. Characterization of Escherichia coli NDM isolates with decreased susceptibility to aztreonam/avibactam: role of a novel insertion in PBP3. J Antimicrob Chemother 70:1420–1428. doi:10.1093/jac/dku568 25634992

[66] Periasamy H, Joshi P, Palwe S, Shrivastava R, Bhagwat S, Patel M. 2020. High prevalence of Escherichia coli clinical isolates in India harbouring four amino acid inserts in PBP3 adversely impacting activity of aztreonam/avibactam. J Antimicrob Chemother 75:1650–1651. doi:10.1093/jac/dkaa021 32040179

[67] Le Terrier C, Nordmann P, Sadek M, Poirel L. 2023*.* In vitro activity of cefepime/zidebactam and cefepime/taniborbactam against aztreonam/avibactam-resistant NDM-like-producing Escherichia coli clinical isolates. J Antimicrob Chemother 78:1191–1194. doi:10.1093/jac/dkad061 36921067 PMC10154122

[68] Mushtaq S, Vickers A, Ellaby N, Woodford N, Livermore DM. 2021. Selection and characterization of mutational resistance to aztreonam/avibactam in β-lactamase-producing Enterobacterales. J Antimicrob Chemother 77:98–111. doi:10.1093/jac/dkab346 34568905

[69] Ma K, Zong Z. 2022. Resistance to aztreonam-avibactam due to CTX-M-15 in the presence of penicillin-binding protein 3 with extra amino acids in Escherichia coli. Front Microbiol 13:1047109. doi:10.3389/fmicb.2022.1047109 36406430 PMC9674307

[70] King AM, Reid-Yu SA, Wang W, King DT, De Pascale G, Strynadka NC, Walsh TR, Coombes BK, Wright GD. 2014. Aspergillomarasmine A overcomes metallo-β-lactamase antibiotic resistance. Nature 510:503–506. doi:10.1038/nature13445 24965651 PMC4981499

[71] Mojica MF, Bonomo RA, Fast W. 2016. B1-metallo-β-lactamases: where do we stand? Curr Drug Targets 17:1029–1050. doi:10.2174/1389450116666151001105622 26424398 PMC4814356

[72] Rotondo CM, Wright GD. 2017. Inhibitors of metallo-β-lactamases. Curr Opin Microbiol 39:96–105. doi:10.1016/j.mib.2017.10.026 29154026

[73] Aoki N, Ishii Y, Tateda K, Saga T, Kimura S, Kikuchi Y, Kobayashi T, Tanabe Y, Tsukada H, Gejyo F, Yamaguchi K. 2010. Efficacy of calcium-EDTA as an inhibitor for metallo-β-lactamase in a mouse model of Pseudomonas aeruginosa pneumonia. Antimicrob Agents Chemother 54:4582–4588. doi:10.1128/AAC.00511-10 20713659 PMC2976153

[74] Yoshizumi A, Ishii Y, Kimura S, Saga T, Harada S, Yamaguchi K, Tateda K, Livermore DM, Woodford N, Livermore DM. 2013. Efficacies of calcium–EDTA in combination with imipenem in a murine model of sepsis caused by Escherichia coli with NDM-1 β-lactamase. J Infect Chemother 19:992–995. doi:10.1007/s10156-012-0528-y 23233082

[75] CLSI. 2012. Methods for dilution antimicrobial susceptibility tests for bacteria that grow aerobically. Approved standard—ninth edition. M07-A9. Clinical and Laboratory Standards Institute, Wayne, PA.

[76] Miles AA, Misra SS, Irwin JO. 1938. The estimation of the bactericidal power of the blood. J Hyg (Lond) 38:732–749. doi:10.1017/s002217240001158x 20475467 PMC2199673

[77] Kondratieva A, Palica K, Frøhlich C, Hovd RR, Leiros H-KS, Erdelyi M, Bayer A. 2024. Fluorinated captopril analogues inhibit metallo-β-lactamases and facilitate structure determination of NDM-1 binding pose. Eur J Med Chem 266:116140. doi:10.1016/j.ejmech.2024.116140 38242072

[78] Lorentzen Øyvind M, Haukefer ASB, Johnsen PJ, Frøhlich C. 2024. The biofilm lifestyle shapes the evolution of β-lactamases. Genome Biol Evol 16:evae030. doi:10.1093/gbe/evae030 38366392 PMC10917518

[79] Lorentzen ØM, Abel S, Johnsen PJ, Frøhlich C. 2025. Biofilm selection constitutively activates c-di-GMP synthesis by the bifunctional enzyme MbaA. bioRxiv. doi:10.1101/2025.02.13.638143

[80] Fröhlich C, Bunzel HA, Buda K, Mulholland AJ, van der Kamp MW, Johnsen PJ, Leiros H-KS, Tokuriki N. 2024. Epistasis arises from shifting the rate-limiting step during enzyme evolution of a β-lactamase. Nat Catal 7:499–509. doi:10.1038/s41929-024-01117-4 38828429 PMC11136654

[81] Christopeit T, Albert A, Leiros H-KS. 2016. Discovery of a novel covalent non-β-lactam inhibitor of the metallo-β-lactamase NDM-1. Bioorg Med Chem 24:2947–2953. doi:10.1016/j.bmc.2016.04.064 27184103

[82] Christopeit T, Carlsen TJO, Helland R, Leiros H-KS. 2015. Discovery of novel inhibitor scaffolds against the metallo-β-lactamase VIM-2 by surface plasmon resonance (SPR) based fragment screening. J Med Chem 58:8671–8682. doi:10.1021/acs.jmedchem.5b01289 26477515

[83] Samuelsen Ø, Castanheira M, Walsh TR, Spencer J. 2008. Kinetic characterization of VIM-7, a divergent member of the VIM metallo-beta-lactamase family. Antimicrob Agents Chemother 52:2905–2908. doi:10.1128/AAC.00166-08 18559652 PMC2493091

