# Supplementary material for: APC24-7, a covalent combination of boronic acid and chelator moieties, restores β-lactam efficiency against metallo-β-lactamase-producers
Source: mSphere. 2025 Dec 3;10(12):e00418-25. doi: 10.1128/msphere.00418-25 (PMC12724231; doi:10.1128/msphere.00418-25)
Supplement: Supplemental material — Protocol S1, Fig. S1 and S2, and Tables S1-S9. [file msphere.00418-25-s0001.docx]

****Supplemental material for****

APC24-7, a Covalent Combination of Boronic Acid and Chelator Moieties Restores β-lactam Efficiency Against Metallo- β-lactamase-Producers

Rebekka Rolfsnes Hovd,^a,b^ Åsmund Kaupang,^b^ Pål Rongved,^a,b^ Geir Kildahl-Andersen,^c^ Knut Tormodssønn Hylland,^c^ Ragnar Hovland,^a^ Ole Andreas Løchen Økstad,^b^ Hanne Cecilie Winther-Larsen,^b^# and Christopher Frøhlich^d^

^a^Adjutec Pharma, Oslo, Norway

^b^Department of Pharmacy, University of Oslo, Oslo, Norway

^c^Kappa solutions, Oslo, Norway

^d^UiT The Arctic University of Norway, Department of Pharmacy, Tromsø, Norway

**Table of contents**

1. Supplementary methods 2

1.1 Protocol S1: General experimental procedures and synthesis of APC24-7 2

2. Supplementary tables 10

Table S1: Overview of bacterial isolates/strains used in this study 10

Table S2: Antimicrobial activity of β-lactamase inhibitors. 12

Table S3: Antimicrobial activity of β-lactam/APC24-7 combinations in clinical isolates of *Escherichia coli* and *Klebsiella pneumoniae* 13

Table S4: Antimicrobial activity of meropenem in combination with APC247/taniborbactam at varying inhibitor concentrations 14

Table S5: Biochemical evaluation of APC24-7. 15

Table S6: Checkerboards for meropenem and isogenic *E. coli* E. cloni 10G 16

Table S7: Checkerboards for cefepime and isogenic *E. coli* E. cloni 10G 16

Table S8: Zn^2+^ susceptibility assays of isogenic *Escherichia coli* 17

Table S9: List of primers constructed and used in this study. 18

3. Supplementary Figures 19

Figure S1. Chemical structure of APC24-7. 19

Figure S2. Concentration-response curves for APC24-7. 20

4. References 21

## Supplementary methods

### Protocol S1: General experimental procedures and synthesis of APC24-7

For synthesis of APC24-7 (Figure S1), all experiments were conducted under N_2_ in anhydrous solvents unless noted otherwise. The following compounds were prepared according to literature methods: *tert-*butyl (2-(bis(pyridin -2-ylmethyl)amino)ethyl)carbamate, (1) and *tert*-butyl 2-((tert-butoxycarbonyl)oxy)-3-((*S*)-2-chloro-2-((3a*S*,4*S*,6*S*,7a*R*)-3a,5,5-trimethylhexahydro-4,6-methanobenzo[d][1,3,2]dioxaborol-2-yl)ethyl)benzoate, (2).

**Synthesis of methyl 4-(((2-(bis(pyridin-2-ylmethyl)amino)ethyl)(tert-butoxycarbonyl)amino)methyl)benzoate (Scheme S1).**

Scheme S1. Synthesis of construct 4-(((2-(bis(pyridin-2-ylmethyl)amino)ethyl)(tert-butoxycarbonyl)amino)methyl)benzoate.

CAUTION: sodium hydride in combination with DMF is susceptible to thermal runaways with low onset temperatures. (3) A solution of *tert-*butyl (2-(bis(pyridin-2-ylmethyl)amino)ethyl)carbamate (5.15 g, 15.1 mmol) in 250 mL dry DMF was cooled down on an ice bath under nitrogen. Sodium hydride (1.81 g 60% dispersion, 45.3 mmol) was added followed by methyl 4-bromomethylbenzoate (3.80 g, 16.6 mmol). After 1 h, the reaction mixture was allowed to reach room temperature and stirred overnight. The reaction mixture was mixed with ethyl acetate (750 mL), the phases were separated, the organic phase was washed with water (250 mL) and the aqueous phase re-extracted with ethyl acetate (250 mL). The combined extracts were washed with water (250 mL) and saturated NaCl (aq), followed by drying over Na_2_SO_4_ (s). Filtration and removal of solvent under reduced pressure gave 6.15 g crude product, which was purified on a dry column vacuum chromatography (DCVC) column, using silica as adsorbent and eluted with a 0%-5% gradient of methanol in dichloromethane. Fractions with product were combined and following evaporation of solvent gave 3.38 g of product (46%) as a light brown oil.

^1^H NMR (400 MHz, chloroform-*d*) δ_H_ 8.49 (m, 2H), 7.91 (d, *J* = 7.9 Hz, 2H), 7.40 (dd, *J* = 7.7 Hz, 1.8 Hz, 1H), 7.62 (m, 2H), 7.51-7.44 (m, 2H), 7.18-7.12 (m, 4H), 4.43-4.31 (m, 2H), 3.89-3.81 (m, 7H), 3.43-3.27 (m, 2H), 2.77-2.66 (m, 2H), 1.34 (br s, 9H). The spectrum is compatible with restricted carbamate rotation.

MS (ESI, positive mode) *m*/*z* 491.3 [M+H]^+^.

**Synthesis of 4-(((2-(bis(pyridin-2-ylmethyl)amino)ethyl)(tert-butoxycarbonyl)amino)methyl)benzoic acid (Scheme S2).**

Scheme S2. Synthesis of 4-(((2-(bis(pyridin-2-ylmethyl)amino)ethyl)(tert-butoxycarbonyl)amino)methyl)benzoic acid.

To an ice-cold solution of methyl 4-(((2-(bis(pyridin-2-ylmethyl)amino)ethyl)(*tert*-butoxycarbonyl)amino)methyl)benzoate (460.4 mg, 0.938 mmol) in 15 mL tetrahydrofuran and water (15 mL), lithium hydroxide monohydrate (157.9 mg, 3.76 mmol) was added. After stirring on an ice bath for 30 min, the mixture was allowed to stir at room temperature overnight, after which the reaction mixture was neutralized with 1 M HCl (aq). After removal of volatiles under reduced pressure, the crude material was used “as is” in the subsequent coupling step.

**Synthesis of tert-Butyl 3-((R)-2-(bis(trimethylsilyl)amino)-2-((3aS,4S,6S,7aR)-3a,5,5-trimethylhexahydro-4,6-methanobenzo[d][1,3,2]dioxaborol-2-yl)ethyl)-2-((tert-butoxycarbonyl)oxy)benzoate (Scheme S3).**

Scheme S3. Synthesis of *tert*-butyl 3-((*R*)-2-(bis(trimethylsilyl)amino)-2-((3a*S*,4*S*,6*S*,7a*R*)-3a,5,5-trimethylhexahydro-4,6-methanobenzo[d][1,3,2]dioxaborol-2-yl)ethyl)-2-((*tert*-butoxycarbonyl)oxy)benzoate.

A flask containing *tert*-butyl 2-((tert-butoxycarbonyl)oxy)-3-((*S*)-2-chloro-2-((3a*S*,4*S*,6*S*,7a*R*)-3a,5,5-trimethylhexahydro-4,6-methanobenzo[d][1,3,2]dioxaborol-2-yl)ethyl)benzoate (624.1 mg, 1.167 mmol) under a nitrogen atmosphere was dissolved in 20 mL tetrahydrofuran and cooled down to -85 °C. A solution of lithium bis(trimethylsilyl)amide, in tetrahydrofuran was added (1.0 M, 1.3 mL, 1.3 mmol) was added, and the solution was then allowed to slowly reach room temperature. Stirring was continued over night, after which volatiles were removed under reduced pressure. The crude product was stirred in *n*-heptane (50 mL) under nitrogen for 30 min and filtered through celite. The filter pad was washed with *n*-heptane (2 x 25 mL) and after removal of solvent under reduced pressure the resulting crude material was immediately reacted in the next step.

**Synthesis of tert-butyl 3-((R)-2-amino-2-((3aS,4S,6S,7aR)-3a,5,5-trimethylhexahydro-4,6-methanobenzo[d][1,3,2]dioxaborol-2-yl)ethyl)-2-((tert-butoxycarbonyl)oxy)benzoate (Scheme S4).**

Scheme S4. Synthesis of *tert*-butyl 3-((*R*)-2-amino-2-((3a*S*,4*S*,6*S,*7a*R*)-3a,5,5-trimethylhexahydro-4,6-methanobenzo[d][1,3,2]dioxaborol-2-yl)ethyl)-2-((*tert*-butoxycarbonyl)oxy)benzoate.

The crude material from the preceding step was dissolved in tetrahydrofuran (20 mL) and put under a nitrogen atmosphere. Methanol (2 mL) was added, and the mixture was stirred at room temperature for 2h. After removal of the solvent, the crude material was immediately used “as is” in the following step.

**Synthesis of tert-butyl 3-((R)-2-(4-(((2-(bis(pyridin-2-ylmethyl)amino)ethyl)(tert-butoxycarbonyl)amino)methyl)benzamido)-2-((3aS,4S,6S,7aR)-3a,5,5-trimethylhexahydro-4,6-methanobenzo[d][1,3,2]dioxaborol-2-yl)ethyl)-2-((tert-butoxycarbonyl)oxy)benzoate (Scheme S5).**

Scheme S5. Synthesis of *tert*-butyl 3-((*R*)-2-(4-(((2-(bis(pyridin-2-ylmethyl)amino)ethyl)(*tert*-butoxycarbonyl)amino)methyl)benzamido)-2-((3a*S*,4*S*,6*S*,7a*R*)-3a,5,5-trimethylhexahydro-4,6-methanobenzo[d][1,3,2]dioxaborol-2-yl)ethyl)-2-((*tert*-butoxycarbonyl)oxy)benzoate.

To 4-(((2-(bis(pyridin-2-ylmethyl)amino)ethyl)(tert-butoxycarbonyl)amino)methyl)benzoic acid (est. 0.938 mmol) HATU was added (388 mg, 1.02 mmol), followed by dichloromethane (20 mL). The resulting suspension was put under a nitrogen atmosphere and cooled down to -20 °C. After dropwise addition of triethylamine (0.2 mL, 1.4 mmol), the mixture was stirred first at -20 °C for 30 min, and then at room temperature for 1 h, before again being cooled to -20 °C. The amine prepared above (est. 1.167 mmol) was then added dropwise as a solution in dichloromethane (10 mL), after which the mixture was slowly allowed to reach room temperature during overnight stirring. The reaction mixture was quenched with water (50 mL) and extracted with ethyl acetate (1 x 100 mL, 2 x 50 mL). The combined extracts were washed with saturated NaCl (aq), dried over MgSO_4_ (s), and filtered. Removal of the solvent under reduced pressure gave 1.30 g of crude material which was purified with DCVC, using Bondesil-C18-OH as packing material. The column was eluted with a stepwise gradient of methanol in water (50% - 100%, in 10% increments). Pure fractions were collected, and after removal of the solvent under reduced pressure the product was obtained as a pale yellow solid (352.6 mg, 39%). MS (ESI, positive mode) *m*/*z* 996.5 [M+Na]^+^.

**Synthesis of (R)-3-(4-(((2-(bis(pyridin-2-ylmethyl)amino)ethyl)amino) methyl)benzamido)-2-hydroxy-3,4-dihydro-2H-benzo[e][1,2]oxaborinine-8-carboxylic acid disodium salt (APC24-7) (Scheme S6).**

Scheme S6. Synthesis of APC24-7.

A solution in dry dichloromethane (25 mL) of the fully protected intermediate prepared above (1.40 g, 1.44 mmol) was cooled down to -78 °C under nitrogen atmosphere. A solution of boron trichloride in dichloromethane (1.0 M, 7.0 mL, 7 mmol) was added dropwise over 10 min after which the reaction mixture was stirred at -78 °C for 1 h. The resulting suspension was allowed to reach room temperature and was quenched with the addition of 20 mL water. The mixture was transferred to a separatory funnel with 300 mL water and washed 3x with diethyl ether (200 mL + 150 mL + 150 mL). The organic phases were back extracted with 50 mL water. The combined aqueous extracts were neutralized with 40 mL 0.5 M NaOH (aq) to pH 7-8. A colourless precipitate was filtered off and the mother liquor was evaporated to give 2.24 g crude product. The crude material was purified on a large SPE column with 17 g of Bondesil C18-OH material, packed by running through pure methanol, 1:1 methanol/water and 1:9 methanol/water. The loading of the material was done by first suspending it in 20 mL 1:9 methanol/water followed by addition of 20 mL 0.5 M NaOH, after which everything entered solution, followed by addition to the column under careful application of vacuum. Elution was done with 25 mL fractions, using the following scheme: 1:9 methanol/water (4 fractions), 2:8 methanol/water (4 fractions), 3:7 methanol/water (2 fractions), 4:6 methanol water (2 fractions). Fractions 5-10 were judged to contain product of acceptable purity and were collected. After removal of the solvent under reduced pressure the product was obtained as a yellow solid (0.63 g, 70%).

^1^H NMR (400 MHz, water-*d_2_*): δ_H_ 8.25 (m, 2H), 7.62 (app. td, *J* = 7.8 Hz, 1.5 Hz, 2H), 7.56 (m, 2H), 7.27 (d, *J* = 7.9 Hz, 2H), 7.19-7.12 (m, 5H), 7.05 (m, 1H), 6.65 (t, *J* = 7.4 Hz, 1H), 3.63 (s, 4H), 3.47 (s, 2H), 3.21 (m, 1H), 3.04 (m, 1H), 2.92 (m, 1H), 2.58 (m, 2H), 2.37 (m, 2H). ^13^C NMR (101 MHz, water-*d_2_*): δ_C_ 178.3, 170.9, 157.7, 153.8, 147.9, 142.5, 137.8, 133.1, 130.6, 128.6, 128.3, 127.0, 125.8, 125.5, 124.1, 122.9, 116.9, 60.4, 53.3, 51.5, 44.6, 39.9 (observed through HSQC only), 32.9 ppm. MS (ESI, positive mode) *m*/*z* 566.3 [M+H]^+^. HRMS (ESI, positive mode): *m*/*z* calcd. for C31H33BN5O5: 566.2569 [M+H]^+^; found 566.2565.

## Supplementary tables

### Table S1: Overview of bacterial isolates/strains used in this study

| **Bacteria** | **Isolate/strain** | **Sequencing type** | **β-lactamase(s)** | **Ref.** |
| --- | --- | --- | --- | --- |
| **Clinical isolates used** | | | | |
| *Escherichia coli* | KresCPE0367 | ST167 | NDM-5, CTX-M-15, OXA-1 | (4) |
| *Escherichia coli* | KresCPE0372 | ST2083 | NDM-5, OXA-181, CMY-42, TEM-1 | (4) |
| *Escherichia coli* | KresCPE0348 | ST38 | OXA-48, CTX-M-24, TEM-1 | (4) |
| *Escherichia coli* | KresCPE0097 | ST10 | KPC-2 | (4) |
| *Klebsiella pneumoniae* | KresCPE0385 | ST101 | OXA-48, CTX-M-15, SHV-1 | (4) |
| *Klebsiella pneumoniae* | KresCPE0353 | ST147 | NDM-1, KPC-2, CTX-M-15, OXA-1, SHV-11 | (4) |
| *Escherichia coli* | #53 | ST95 | IMP-26, CTX-M-15, TEM-1 | (5) |
| *Escherichia coli* | #36 | ST101 | NDM-7, CTX-M-15, OXA-1 | (5) |
| *Escherichia coli* | #50 | ST410 | VIM-4, CTX-M-15, CMY-4, TEM-169 | (5) |
| *Klebsiella pneumoniae* | #22 | ST525 | NDM-1, OXA-181, SHV-11, CTX-M-15, OXA-1, TEM-1 | (5) |
| *Klebsiella pneumoniae* | K66-45 | ST11 | NDM-1, SHV-11, CTX-M-15, OXA1, OXA-9, TEM-1 | (5) |
| *Klebsiella pneumoniae* | #29 | ST11 | NDM-1, SHV-11, CTX-M-15, OXA-1, CMY-6 | (5) |
| *Klebsiella pneumoniae* | K46-62 | ST2134 | VIM-1, SHV-12, TEM-1 | (5) |
| *Klebsiella pneumoniae* | 103973 | ST307 | CTX-M-15 | (6) |
| *Escherichia coli* | 30348 | ST131 | CTX-M-15 | (7) |
| *Escherichia coli* | BAA-2469^™^ | 1001728 | NDM-1, OXA-1, AmpC | ATCC |
| *Klebsiella pneumoniae* | BAA-1705^™^ | ART2008133 | KPC-2, SHV-1, TEM, OXA-18 | ATCC |
| *Klebsiella pneumoniae* | BAA-2146^™^ | 1000527,7561 | NDM-1, SHV-1, CTX-M-1, TEM, OXA-1, AmpC | ATCC |
| *Escherichia coli* | ATCC®25922 | NCIB 12210 | - | ATCC |
| **Isogenic strains constructed** | | | | |
| *Escherichia coli* | MP21-05 | E. cloni 10G | - | Lucigen |
| *Escherichia coli* | MP32-64 | E, cloni 10G | vector control | Lucigen |
| *Escherichia coli* | MP21-01 | E. cloni 10G | OXA-48^a^ | (8) |
| *Escherichia coli* | MP24-44 | E. cloni 10G | KPC-2^a^ | (8) |
| *Escherichia coli* | MP24-80 | E. cloni 10G | CTX-M-15 ^a^ | (8) |
| *Escherichia coli* | MP29-27 | E. cloni 10G | OXA-163 ^a^ | This study |
| *Escherichia coli* | MP30-13 | E. cloni 10G | NDM-4 (NDM-1: M154L) ^a^ | This study |
| *Escherichia coli* | MP30-15 | E. cloni 10G | NDM-9 (NDM1: E152K) ^a^ | This study |
| *Escherichia coli* | MP30-20 | E. cloni 10G | NDM-7 (NDM-1: M154L/D130N) ^a^ | This study |
| *Escherichia coli* | MP30-22 | E. cloni 10G | NDM-16 (NDM-1: V88L/M154L/A233V) ^a^ | This study |
| *Escherichia coli* | MP30-57 | E. cloni 10G | VIM-2 ^a^ | (9) |
| *Escherichia coli* | MP30-58 | E. cloni 10G | IMP-26 ^a^ | (10) |
| *Escherichia coli* | MP30-63 | E. cloni 10G | NDM-1 ^a^ | (8) |
| **Strains used for enzyme expression and purification** | | | | |
| *Escherichia coli* | - | BL21 (DE3) | - | New England Biolabs |
| *Escherichia coli* | - | BL21 (DE3) | NDM-9 | This study |

^a^All β-lactamase were sub-cloned into a low to medium copy number plasmid (origin, pA15)

###

### Table S2: Antimicrobial activity of β-lactamase inhibitors.

The minimum inhibitory concentration (MIC) of APC24-7, APC148 and taniborbactam were determined against a selection of clinical isolates.

| Bacteria | Strain/Isolate | Carbapenemase | Non-carbapenemase | MIC  APC24-7 (μM) |
| --- | --- | --- | --- | --- |
| *E. coli* | 30348 | None | CTX-M-15 | >906 |
| *K. pneumoniae* | 103973 | None | CTX-M-15 | >906 |
| *K. pneumoniae* | BAA1705™ | KPC-2 | SHV-1, TEM, OXA-18 | >906 |
| *E. coli* | BAA-2469™ | NDM-1 | OXA-1, AmpC | >906 |
| *K. pneumoniae* | BAA-2146™ | NDM-1 | SHV-1, CTX-M-1, TEM, OXA-1, AmpC | >906 |
| *K. pneumoniae* | K46-62 | VIM-1 | SHV-12, TEM-1 | >906 |
| *E. coli* | #50 | VIM-4 | CTX-M-15, CMY-4, TEM-169 | >906 |
| *E. coli* | #53 | IMP-26 | CTX-M-15, TEM-1 | >906 |
| *E. coli* | MP21-05 | - | - | >906 |
| *E. coli* | MP32-64 | - | - | >906 |
| APC148^*^ | | | | |
| *E. coli* | MP21-05 | - | - | >906 |
| *E. coli* | MP32-64 | -- | - | >906 |
| Taniborbactam^**^ | | | | |
| *E. coli* | MP21-05 | - | - | >906 |
| *E. coli* | MP32-64 | - | - | >906 |

^*^For antibacterial effect beyond the isogenic strains investigated here, see Samuelsen et al. (11)

^**^For antibacterial effect beyond the isogenic strains investigated here, see Hamrick et al. (12)

### Table S3: Antimicrobial activity of β-lactam/APC24-7 combinations in clinical isolates of *Escherichia coli* and *Klebsiella pneumoniae*

The minimum inhibitory concentration (MIC) for aztreonam (AZT), ceftazidime (CAZ), cefepime (FEP) and amoxicillin (AMX), when tested in combination with APC24-7 against clinical isolates. APC24-7 was fixed at 57 μM for all β-lactam/APC24-7 combinations.

| Bacteria | Strain/Isolate | β-lactamase(s) | MIC AZT (mg/L) | | MIZ CAZ (mg/L) | | MIC FEP (mg/L) | | MIC AMX (mg/L) | |
| --- | --- | --- | --- | --- | --- | --- | --- | --- | --- | --- |
|  |  |  | **AZT alone** | **APC24-7^a^** | **CAZ alone** | **APC24-7^a^** | **FEP alone** | **APC24-7^a^** | **AMX alone** | **APC24-7^a^** |
| *E. coli* | KresCPE0097 | KPC-2 | 64 | 0.25 | 8 | 0.5 | 16 | 0.06 | >64 | 16 |
| *K. pneumoniae* | BAA1705 | KPC-2, SHV-1, TEM, OXA-18 | >64 | 8 | >64 | 8 | >64 | 1 | >64 | >64 |
| *E. coli* | KresCPE0348 | OXA-48, CTX-M-24, TEM-1 | 64 | 0.25 | 4 | 1 | >64 | 1 | >64 | >64 |
| *K. pneumoniae* | KresCPE0385 | OXA-48, CTX-M-15, SHV-1 | >64 | 16 | >64 | 4 | >64 | 8 | >64 | >64 |
| *E. coli* | BAA-2469 | NDM-1, OXA-1, AmpC | 16 | 0.12 | >64 | 16 | >64 | 2 | >64 | >64 |
| *K. pneumoniae* | BAA-2146 | NDM-1, SHV-1, CTX-M-1, TEM, OXA-1, AmpC | >64 | 2 | >64 | 32 | >64 | 4 | >64 | >64 |
| *K. pneumoniae* | K66-45 | NDM-1, SHV-11, CTX-M-15, OXA1, OXA-9, TEM-1 | >64 | 2 | >64 | 64 | >64 | 2 | >64 | >64 |
| *K. pneumoniae* | #29 | NDM-1, SHV-11, CTX-M-15, OXA-1, CMY-6 | >64 | 2 | >64 | 16 | >64 | 2 | >64 | >64 |
| *E. coli* | KresCPE0367 | NDM-5, CTX-M-15, OXA-1 | >64 | 8 | >64 | >64 | >64 | 64 | >64 | >64 |
| *E. coli* | #36 | NDM-7, CTX-M-15, OXA-1 | >64 | 8 | >64 | >64 | >64 | >64 | >64 | >64 |
| *K. pneumoniae* | K46-62 | VIM-1, SHV-12, TEM-1 | >64 | 4 | >64 | 8 | >64 | 0.25 | >64 | >64 |
| *E. coli* | #50 | VIM-4, CTX-M-15, CMY-4, TEM-169 | >64 | 8 | >64 | 4 | >64 | 0.25 | >64 | >64 |
| *E. coli* | #53 | IMP-26, CTX-M-15, TEM-1 | 32 | 0.06 | >64 | 32 | >64 | 1 | >64 | 16 |
| *K. pneumoniae* | KresCPE0353 | NDM-1, KPC-2, CTX-M-15, OXA-1, SHV-11 | >64 | 4 | >64 | 64 | >64 | 2 | >64 | >64 |
| *K. pneumoniae* | #22 | NDM-1, OXA-181, SHV-11, CTX-M-15, OXA-1, TEM-1 | >64 | 2 | >64 | 64 | >64 | 8 | >64 | >64 |
| *E. coli* | KresCPE0372 | NDM-5, OXA-181, CMY-42, TEM-1 | 32 | 8 | >64 | >64 | >64 | 64 | >64 | >64 |

^a^Inhibitor concentrations fixed at 57 μM.

### Table S4: Antimicrobial activity of meropenem in combination with APC247/taniborbactam at varying inhibitor concentrations

The minimum inhibitory concentration (MIC) of meropenem (MEM) was determined in combination with APC24-7 and taniborbactam (TAN) against clinical isolates of *E. coli* and *K. pneumoniae*. Inhibitor concentrations were fixed at 14, 28 and 57 μM.

|  | | |  | MIC MEM/APC24-7 (mg/L) | | | MIC MEM/TAN (mg/L) | | |
| --- | --- | --- | --- | --- | --- | --- | --- | --- | --- |
| Bacteria | **Isolate** | **β-lactamase(s)** | **MIC**  **MEM alone** | **14 μM** | **28 μM** | **57 μM** | **14 μM** | **28 μM** | **57 μM** |
| *E. coli* | KresCPE0097 | KPC-2 | 1-2 | <0.03 | <0.03 | <0.03 | <0.03 | <0.03 | <0.03 |
| *K. pneumoniae* | BAA1705 | KPC-2, SHV-1, TEM, OXA-18 | 32 | 1 | 0.5 | 0.25 | ≤0.03 | ≤0.03 | ≤0.03 |
| *E. coli* | KresCPE0348 | OXA-48, CTX-M-24, TEM-1 | >64 | >64 | 64 | 64 | 4 | 4 | 2 |
| *K. pneumoniae* | KresCPE0385 | OXA-48, CTX-M-15, SHV-1 | 16 | 32 | 16 | 16 | 1 | 2 | 0.5 |
| *E. coli* | BAA-2469 | NDM-1, OXA-1, AmpC | 32-64 | 0.5 | 0.12 | ≤0.03 | 0.12 | 0.125 | 0.06 |
| *K. pneumoniae* | BAA-2146 | NDM-1, SHV-1, CTX-M-1, TEM, OXA-1, AmpC | >64 | 32 | 1 | 0.12 | 8 | 4 | 2 |
| *K. pneumoniae* | K66-45 | NDM-1, SHV-11, CTX-M-15, OXA1, OXA-9, TEM-1 | 32-64 | 8 | 0.5 | 0.06 | 0.5 | 0.25 | 0.25 |
| *K. pneumoniae* | #29 | NDM-1, SHV-11, CTX-M-15, OXA-1, CMY-6 | 64 | 16 | 8 | 0.5 | 1 | 0.5 | 0.25 |
| *E. coli* | KresCPE0367 | NDM-5, CTX-M-15, OXA-1 | >64 | 16 | 8 | 2 | 0.5 | 0.5 | 0.25 |
| *E. coli* | #36 | NDM-7, CTX-M-15, OXA-1 | >64 | 32 | 16 | 4 | 8 | 4 | 2 |
| *K. pneumoniae* | K46-62 | VIM-1, SHV-12, TEM-1 | 32-64 | 1 | 0.25 | 0.06 | 0.06 | 0.06 | 0.06 |
| *E. coli* | #50 | VIM-4, CTX-M-15, CMY-4, TEM-169 | 16-32 | 2 | 0.12 | ≤0.03 | 0.06 | 0.06 | 0.06 |
| *E. coli* | #53 | IMP-26, CTX-M-15, TEM-1 | 8-16 | 8 | 4 | 0.25 | 16 | 16 | 16 |
| *K. pneumoniae* | KresCPE0353 | NDM-1, KPC-2, CTX-M-15, OXA-1, SHV-11 | >64 | 8 | 2 | 0.25 | 0.5 | 0.25 | 0.25 |
| *K. pneumoniae* | #22 | NDM-1, OXA-181, SHV-11, CTX-M-15, OXA-1, TEM-1 | >64 | >64 | 64 | 32 | 32 | 16 | 8 |
| *E. coli* | KresCPE0372 | NDM-5, OXA-181, CMY-42, TEM-1 | >64 | 8 | 4 | 1 | 2 | 0.5 | 0.25 |

###

### Table S5: Biochemical evaluation of APC24-7.

The 50% inhibitory concentrations (*IC*_50_) of APC24-7 were determined against a selection of MBLs (NDM-1, NDM-9, VIM-2, VIM-7, SHD-1, MYO-1 and ECV-1) and the SBL OXA-48. The associated curve-fit parameters are given (see Figure S2 for dose-response curves) and the -log(*IC*_50_) calculated (p*IC*_50_). (13)

| **Enzyme** | **Enzyme conc. (nM)** | **Max inhibitor (µM)** | **p*IC*_50_** | ***IC*_50_ (μM)** | **95% CI** | **Hillslope** | ***R*^2^** |
| --- | --- | --- | --- | --- | --- | --- | --- |
| NDM-1 | 10 | 0.8 | 6.9 | 0.14 | 0.13 to 0.15 | -4.49 | 0.95 |
| NDM-9 | 10 | 125 | 5.6 | 2.66 | 2.05 to 3.45 | -0.84 | 0.86 |
| VIM-2 | 1 | 250 | 7.0 | 0.11 | 0.11 to 0.12 | -1.99 | 0.99 |
| VIM-7 | 1 | 250 | 6.0 | 1.08 | 0.96 to 1.21 | -0.95 | 0.98 |
| SHD-1 | 10 | 1000 | 7.3 | 0.06 | 0.05 to 0.07 | -1.01 | 0.99 |
| ECV-1 | 10 | 100 | 6.8 | 0.17 | 0.15 to 0.19 | -1.13 | 0.96 |
| MYO-1 | 10 | 200 | 7.1 | 0.08 | 0.07 to 0.08 | -1.84 | 0.98 |
| OXA-48 | 1 | 1000 | 6.4 | 0.42 | 0.40 to 0.45 | -1.35 | 0.99 |

### Table S6: Checkerboards for meropenem and isogenic *E. coli* E. cloni 10G

Inhibitors taniborbactam (TAN), APC24-7 and APC148 were tested at an equimolar concentration (left most column) and the minimum inhibitory concentration (MIC) of meropenem (MEM) given for the respective inhibitor concentrations.

|  | MIC MEM/TAN (mg/L) | | MIC MEM/APC24-7 (mg/L) | | MIC MEM/APC148 (mg/L) | |
| --- | --- | --- | --- | --- | --- | --- |
| Inhibitor (μM) | **MP30-58 (IMP-26)** | **MP30-15 (NDM-9)** | **MP30-58 (IMP-26)** | **MP30-15 (NDM-9)** | **MP30-58 (IMP-26)** | **MP30-15 (NDM-9)** |
| 0 | >8 | >8 | >8 | >8 | >8 | >8 |
| 2 | >8 | >8 | >8 | >8 | >8 | >8 |
| 4 | >8 | >8 | >8 | >8 | >8 | >8 |
| 9 | >8 | >8 | >8 | 8 | >8 | >8 |
| 17 | >8 | >8 | >8 | 4 | 0.5 | 0.25 |
| 35 | >8 | 8 | 1 | 0.5 | 0.25 | 0.25 |
| 69 | >8 | 4 | 0.125 | 0.5 | 0.25 | 0.25 |
| 139 | >8 | 4 | 0.06 | 0.03 | 0.25 | 0.25 |

### Table S7: Checkerboards for cefepime and isogenic *E. coli* E. cloni 10G

Inhibitors taniborbactam (TAN), APC24-7 and APC148 were tested at an equimolar concentration and the minimum inhibitory concentration (MIC) of cefepime (FEP) given for the respective inhibitor concentrations.

|  | MIC FEP/TAN (mg/L) | | MIC FEP/APC24-7 (mg/L) | | MIC FEP/APC148 (mg/L) | |
| --- | --- | --- | --- | --- | --- | --- |
| Inhibitor (μM) | **MP30-58 (IMP-26)** | **MP30-15 (NDM-9)** | **MP30-58 (IMP-26)** | **MP30-15 (NDM-9)** | **MP30-58 (IMP-26)** | **MP30-15 (NDM-9)** |
| 0 | >8 | >8 | >8 | >8 | >8 | >8 |
| 2 | >8 | >8 | >8 | >8 | >8 | >8 |
| 4 | >8 | >8 | >8 | >8 | >8 | >8 |
| 9 | >8 | >8 | >8 | >8 | >8 | >8 |
| 17 | >8 | >8 | >8 | 4 | 1 | 1 |
| 35 | >8 | >8 | 2 | 1 | 1 | 1 |
| 69 | >8 | >8 | 1 | 1 | 1 | 1 |
| 139 | >8 | >8 | 1 | 1 | 1 | 1 |

### Table S8: Zn^2+^ susceptibility assays of isogenic *Escherichia coli*

Minimum inhibitory concentration (MIC) of meropenem (MEM) in combination with APC24-7, APC148 and taniborbactam (TAN) for isogenic *E. coli* E. cloni 10G producing MBLs NDM-9, IMP-26, NDM-1 or VIM-2. MIC was tested in Mueller-Hinton Broth (MHB) and in MHB supplemented with 100 μM Zn^2+^.

|  | MIC MEM/Inhibitor (mg/L) | | | | | | | |
| --- | --- | --- | --- | --- | --- | --- | --- | --- |
|  | **MP30-15**  **(NDM-9)** | | **MP30-58**  **(IMP-26)** | | **MP30-63**  **(NDM-1)** | | **MP30-57**  **(VIM-2)** | |
| Inhibitor^b^ | **MHB** | **MHB +100 μM Zn^2+^** | **MHB** | **MHB +100 μM Zn^2+^** | **MHB** | **MHB +100 μM Zn^2+^** | **MHB** | **MHB +100 μM Zn^2+^** |
| None | >64 | >64 | 32 | 64 | >64 | >64 | 16 | 16 |
| APC148 | 0.5 | >64 | 0.25 | 64 | 0.06 | >64 | 1 | 16 |
| APC24-7 | 8 | >64 | 4 | 64 | 0.12 | 0.12 | 1 | 0.5 |
| TAN | >64 | >64 | 32 | 64 | 1 | 1 | 1 | 0.5 |

^b^Inhibitor concentrations were fixed at 35 μM for NDM-1, NDM-9 and VIM-2 and 70 μM for IMP-26.

### Table S9: List of primers constructed and used in this study.

| **No.** | **Description** |  | **5’ - 3’** | **Reference** |
| --- | --- | --- | --- | --- |
| CF80 | *NDM_M154L* | F | TTTTTTGCTCTTCGCAAGAGGGGCTGGTTGCGGCGC | This study |
| CF80 | *NDM_M154L* | R | TTTTTTGCTCTTCCTTGCGGGGCAAGCTGGTTCGA | This study |
| CF84 | *NDM_D130N* | F | TTTTTTGCTCTTCGGGCGGTATGAATGCGCTGCATG | This study |
| CF84 | *NDM_D130N* | R | TTTTTTGCTCTTCCGCCCATCTTGTCCTGATGCGCG | This study |
| CF86 | *NDM_E152K* | F | TTTTTTGCTCTTCTGCCCCGCAAAAGGGGATGGTTG  TTTTTTGCTCTTC GGGCA AGCTGGTTCGACAACGCA | This study |
| CF86 | *NDM_E152K* | R | TTTTTTGCTCTTCTGCCCCGCAAAAGGGGATGGTTG  TTTTTTGCTCTTC GGGCA AGCTGGTTCGACAACGCA | This study |
| CF82 | NDM_V88L | F | TTTTTTGCTCTTCCCGCGTGCTGCTGGTCGATACCG | This study |
| CF82 | NDM_V88L | R | TTTTTTGCTCTTCCGCGGCCGCCATCCCTG | This study |
| CF83 | NDM_A233V | F | TTTTTTGCTCTTCCGCCGCGTCAGTTCGCGCGTTTG  TTTTTTGCTCTTC CGGCG TAGTGCTCAGTGTCGGC | This study |
| CF83 | NDM_A233V | R | TTTTTTGCTCTTCCGCCGCGTCAGTTCGCGCGTTTG  TTTTTTGCTCTTC CGGCG TAGTGCTCAGTGTCGGC | This study |
| CF54 | *OXA-163* | F | TTTTGCTCTTCTATTCGGGCTAAAACTGGATACGATACTAAGATTGGCTGG | This study |
| CF54 | *OXA-163* | R | TTTTTGCTCTTCGAATAATATAGTCGCCATTG | This study |
| CF7 | *preSeq* | F | GATTACGCGCAGACCAAAACG | (8) |
| CF8 | *postSeq* | R | CCTATTTCCCTAAAGGGTTTATTGAGAATATG | (8) |
| P1 | T7-promoter-Fw | F | TAATACGACTCACTATAGGG | Thermo Fisher Scientific |
| P2 | T7-promoter-Rv | R | GCTAGTTATTGCTCAGCGG | Thermo Fisher Scientific |
| P3 | Gibson-NDM9 | F | AGAACCTGTATTTTCAGGGTGGTGAAATCCGCCCGACG | This study |
| P4 | Gibson-NDM9 | R | GAATCACTCGAGAGGCACTTTCAGCGCAGCTTGTCGGC | This study |
| P5 | Gibson-pOML213-NDM9 | F | TGGCCGACAAGCTGCGCTGAAAGTGCCTCTCGAGTGATTCG | This study |
| P6 | Gibson-pOML213-NDM9 | R | ATCGTCGGGCGGATTTCACCACCCTGAAAATACAGGTTCTCTGCGG | This study |

## Supplementary Figures

### Figure S1. Chemical structure of APC24-7.

The chemical structure of APC24-7. The general experimental procedures and synthesis of APC24-7 is shown in protocol S1.


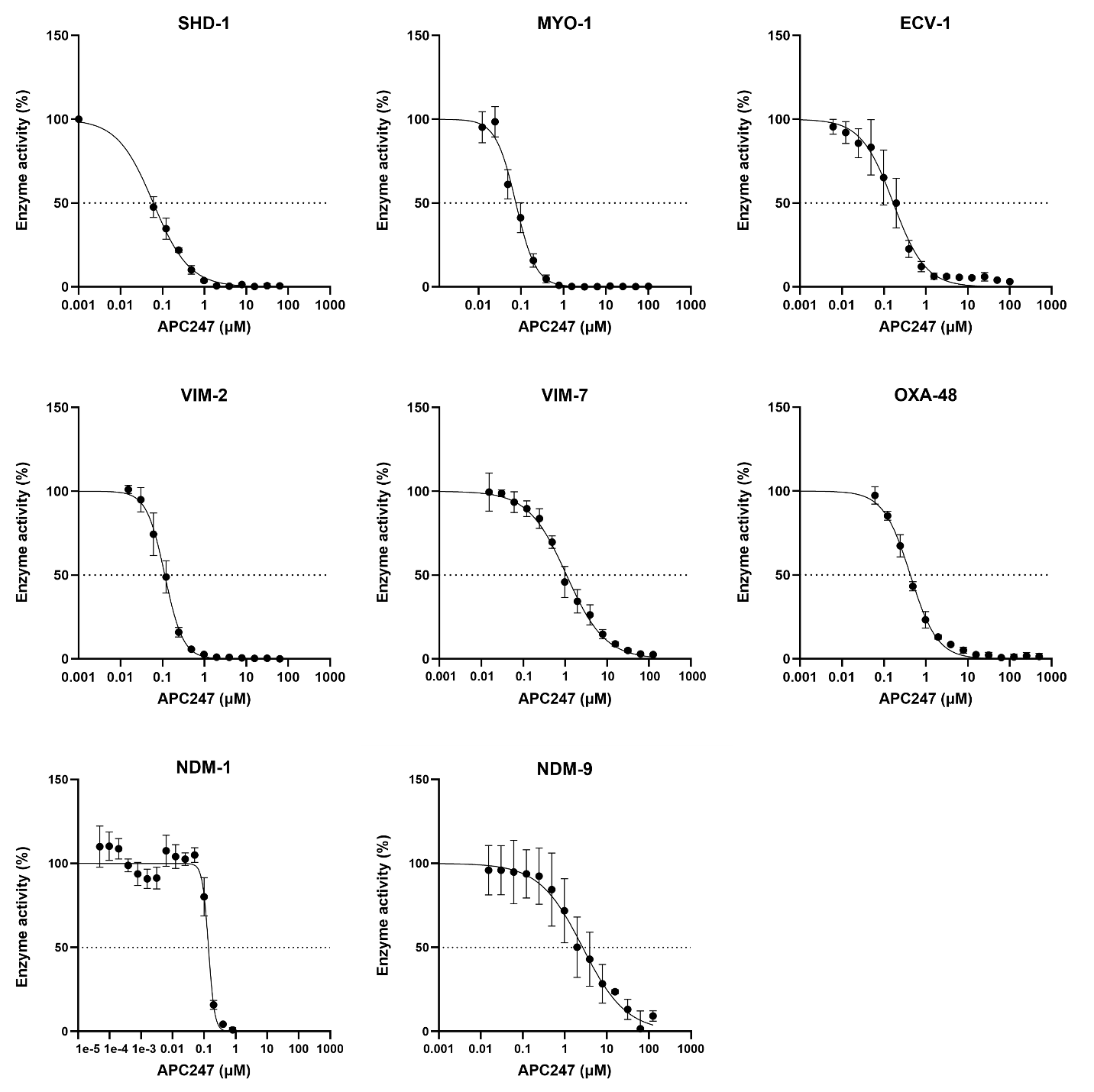


### Figure S2. Concentration-response curves for APC24-7.

The concentration-dependent inhibitory curves for APC24-7 toward various MBLs (SHD-1, MYO-1, ECV-1, VIM-2, VIM-7, OXA-48, NDM-1 and NDM-9) and the SBL OXA-48. The solid lines show the best-fit curve and dotted lines indicate 50% enzyme activity (*IC*_50_). Errors represent the standard deviation based on at least 4 replicates. Determined *IC*_50_ values and curve-fitting parameters are reported in Table S5. The residual enzyme activity was calculated by analyzing initial velocities compared to [APC24-7] = 0.

## References

1. Kawabata E, Kikuchi K, Urano Y, Kojima H, Odani A, Nagano T. 2005. Design and synthesis of zinc-selective chelators for extracellular applications. J Am Chem Soc 127:818-9.

2. Hecker SJ, Reddy KR, Lomovskaya O, Griffith DC, Rubio-Aparicio D, Nelson K, Tsivkovski R, Sun D, Sabet M, Tarazi Z, Parkinson J, Totrov M, Boyer SH, Glinka TW, Pemberton OA, Chen Y, Dudley MN. 2020. Discovery of Cyclic Boronic Acid QPX7728, an Ultrabroad-Spectrum Inhibitor of Serine and Metallo-β-lactamases. J Med Chem 63:7491-7507.

3. Yang Q, Sheng M, Henkelis JJ, Tu S, Wiensch E, Zhang H, Zhang Y, Tucker C, Ejeh DE. 2019. Explosion Hazards of Sodium Hydride in Dimethyl Sulfoxide, N,N-Dimethylformamide, and N,N-Dimethylacetamide. Organic Process Research & Development 23:2210-2217.

4. Ljungquist O, Haldorsen B, Pontinen AK, Janice J, Josefsen EH, Elstrom P, Kacelnik O, Norwegian Study Group on CPE, Sundsfjord A, Samuelsen O, Members of The Norwegian Study Group on CPE. 2023. Nationwide, population-based observational study of the molecular epidemiology and temporal trend of carbapenemase-producing Enterobacterales in Norway, 2015 to 2021. Euro Surveill 28:2200774.

5. Samuelsen O, Overballe-Petersen S, Bjornholt JV, Brisse S, Doumith M, Woodford N, Hopkins KL, Aasnaes B, Haldorsen B, Sundsfjord A, Norwegian Study Group on CPE. 2017. Molecular and epidemiological characterization of carbapenemase-producing Enterobacteriaceae in Norway, 2007 to 2014. PLoS One 12:e0187832.

6. Fostervold A, Hetland MAK, Bakksjo R, Bernhoff E, Holt KE, Samuelsen O, Simonsen GS, Sundsfjord A, Wyres KL, Lohr IH, Norwegian Study Group on Klebsiella p. 2022. A nationwide genomic study of clinical *Klebsiella pneumoniae* in Norway 2001-15: introduction and spread of ESBLs facilitated by clonal groups CG15 and CG307. J Antimicrob Chemother 77:665-674.

7. Gladstone RA, McNally A, Pontinen AK, Tonkin-Hill G, Lees JA, Skyten K, Cleon F, Christensen MOK, Haldorsen BC, Bye KK, Gammelsrud KW, Hjetland R, Kummel A, Larsen HE, Lindemann PC, Lohr IH, Marvik A, Nilsen E, Noer MT, Simonsen GS, Steinbakk M, Tofteland S, Vattoy M, Bentley SD, Croucher NJ, Parkhill J, Johnsen PJ, Samuelsen O, Corander J. 2021. Emergence and dissemination of antimicrobial resistance in *Escherichia coli* causing bloodstream infections in Norway in 2002-17: a nationwide, longitudinal, microbial population genomic study. Lancet Microbe 2:e331-e341.

8. Fröhlich C, Sorum V, Tokuriki N, Johnsen PJ, Samuelsen O. 2022. Evolution of β-lactamase-mediated cefiderocol resistance. J Antimicrob Chemother 77:2429-2436.

9. Lorentzen OM, Haukefer ASB, Johnsen PJ, Frohlich C. 2024. The Biofilm Lifestyle Shapes the Evolution of β-Lactamases. Genome Biol Evol 16.

10. Kondratieva A, Palica K, Frohlich C, Hovd RR, Leiros HS, Erdelyi M, Bayer A. 2024. Fluorinated captopril analogues inhibit metallo-β-lactamases and facilitate structure determination of NDM-1 binding pose. Eur J Med Chem 266:116140.

11. Samuelsen O, Astrand OAH, Frohlich C, Heikal A, Skagseth S, Carlsen TJO, Leiros HS, Bayer A, Schnaars C, Kildahl-Andersen G, Lauksund S, Finke S, Huber S, Gjoen T, Andresen AMS, Okstad OA, Rongved P. 2020. ZN148 Is a Modular Synthetic Metallo-β-Lactamase Inhibitor That Reverses Carbapenem Resistance in Gram-Negative Pathogens In Vivo. Antimicrob Agents Chemother 64:e02415-19.

12. Hamrick JC, Docquier JD, Uehara T, Myers CL, Six DA, Chatwin CL, John KJ, Vernacchio SF, Cusick SM, Trout REL, Pozzi C, De Luca F, Benvenuti M, Mangani S, Liu B, Jackson RW, Moeck G, Xerri L, Burns CJ, Pevear DC, Daigle DM. 2020. VNRX-5133 (Taniborbactam), a Broad-Spectrum Inhibitor of Serine- and Metallo-β-Lactamases, Restores Activity of Cefepime in Enterobacterales and *Pseudomonas aeruginosa*. Antimicrob Agents Chemother 64:10.1128/aac.01963-19.

13. Thakur A, Kumar A, Sharma V, Mehta V. 2022. PIC50: An open source tool for interconversion of PIC50 values and IC50 for efficient data representation and analysis. bioRxiv doi:10.1101/2022.10.15.512366:2022.10.15.512366.
